# Recent Advances in Application of Alkoxy Radical in Organic Synthesis

**DOI:** 10.3390/org4040033

**Published:** 2023-09-28

**Authors:** Munsaf Ali, Shi Sewell, Juncheng Li, Ting Wang

**Affiliations:** Department of Chemistry, University at Albany, State University of New York, 1400 Washington Ave., Albany, NY 12222, USA

**Keywords:** alkoxy radicals, photoredox, natural product synthesis

## Abstract

Alkoxy radicals have been identified as versatile intermediates in synthetic chemistry in the last few decades. Over the last decade, various catalytic processes for the in situ generation of alkoxy radicals have been explored, leading to the development of new synthetic methodologies based on alkoxy radicals. In this review, we provided a comprehensive review of recent developments in the utilization of alkoxy radicals in diverse organic transformations, natural product synthesis, and the late-stage modification of bioactive molecules through the implementation of the photoredox methodology.

## 1. Introduction

Alkoxy radicals are highly reactive structural frameworks in which radicals are localized at the oxygen atom and singularly bound to an alkyl group [1,2]. Alkoxy radicals, without stabilization by delocalization effects, are a highly energetic class of intermediates among heteroatom-centered radicals. Modern catalytic methods utilize oxygen-centered radicals as versatile intermediates [3–6]. Alkoxy radical intermediates deliver the most stabilized alkyl radical via the β-scission pathway, resulting in unsymmetrical substrates that are highly expected [7–9]. This radical chemistry has been well utilized for natural product synthesis in recent decades [10–14]. However, there are still many challenges in in situ generating oxygen radical species from free alcohols due to the lower reduction potential and strong bond dissociation energy (BDE, ~105 kcal mol^−1^) of the alcoholic bond [15]. These barriers hinder their application in synthetic organic transformations. The O-radicals can be indirectly generated from conventional sources such as lead (IV) alkoxides [16], peroxides [17,18], sulfonates [19,20], hypohalites nitrite esters [12,21], *N*-alkoxylpyridine-2-thiones [22], and *N*-alkoxy phthalimides [13,23,24].

**Scheme 1.**
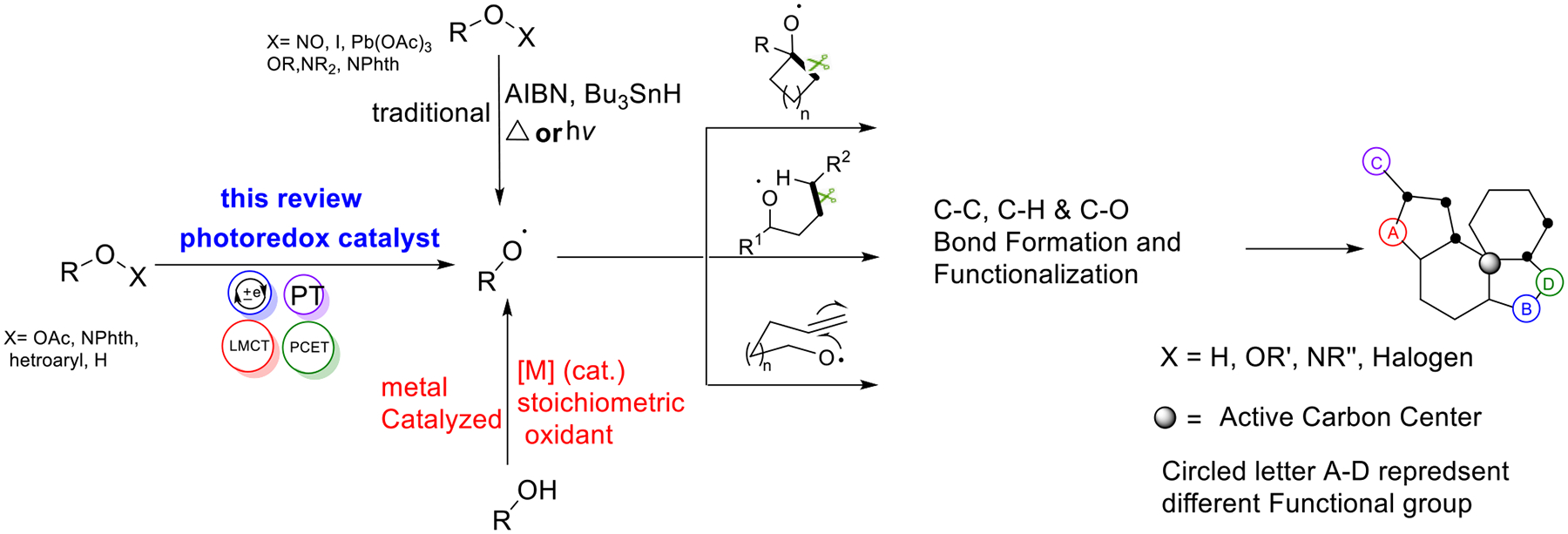
Alkoxy radical promoted organic transformation for natural motifs.

The development of radical chemistry via photoredox catalysis has facilitated a wide range of applications in organic transformation over recent years [25]. This novel approach is gaining high interest because it produces highly energetic radical intermediates that are difficult to obtain through conventional catalytic methodologies. It has established remarkable progress in carbon radical generation, realizing new C-X, C-C, C-N, C-H, and C-S bond formation [26–28]. Nitrogen and oxygen-centered radicals have also been widely applied in organic synthesis [2,4,29,30]. The light-induced catalytic system has been an attractive field where synthetic chemists could harness solar energy to generate the desired radical species [31–33], especially using heteroatom-centered radicals [34–38]. (Scheme 1). In addition to the well-discovered MLCT of coordination complexes [39,40] and organic promoters [2,41,42], electron excitation processes supported by redox complexes [43], novel PCET process [44], and unexpected excitations of organometallic compounds were also applied as efficient catalytic models to trigger photoactivation [5,6,45]. Such photocatalysts have been used for challenging applications, such as getting clean energy fuel by the splitting of water molecules [46,47] and reducing carbon dioxide into methane [48,49].

The recent synthetic method development of alkoxy radicals has promoted various applications. This review focuses on the recent development of alkoxy radical generation and natural product syntheses by using alkoxy radicals as key intermediates. In the first part, the recent development of alkoxy radical generation methods will be summarized in chronological order; the second part will present the recent applications of alkoxy radicals in natural product syntheses.

## 2. Photocatalytic Generation of Alkoxy Radicals

**Scheme 2.**
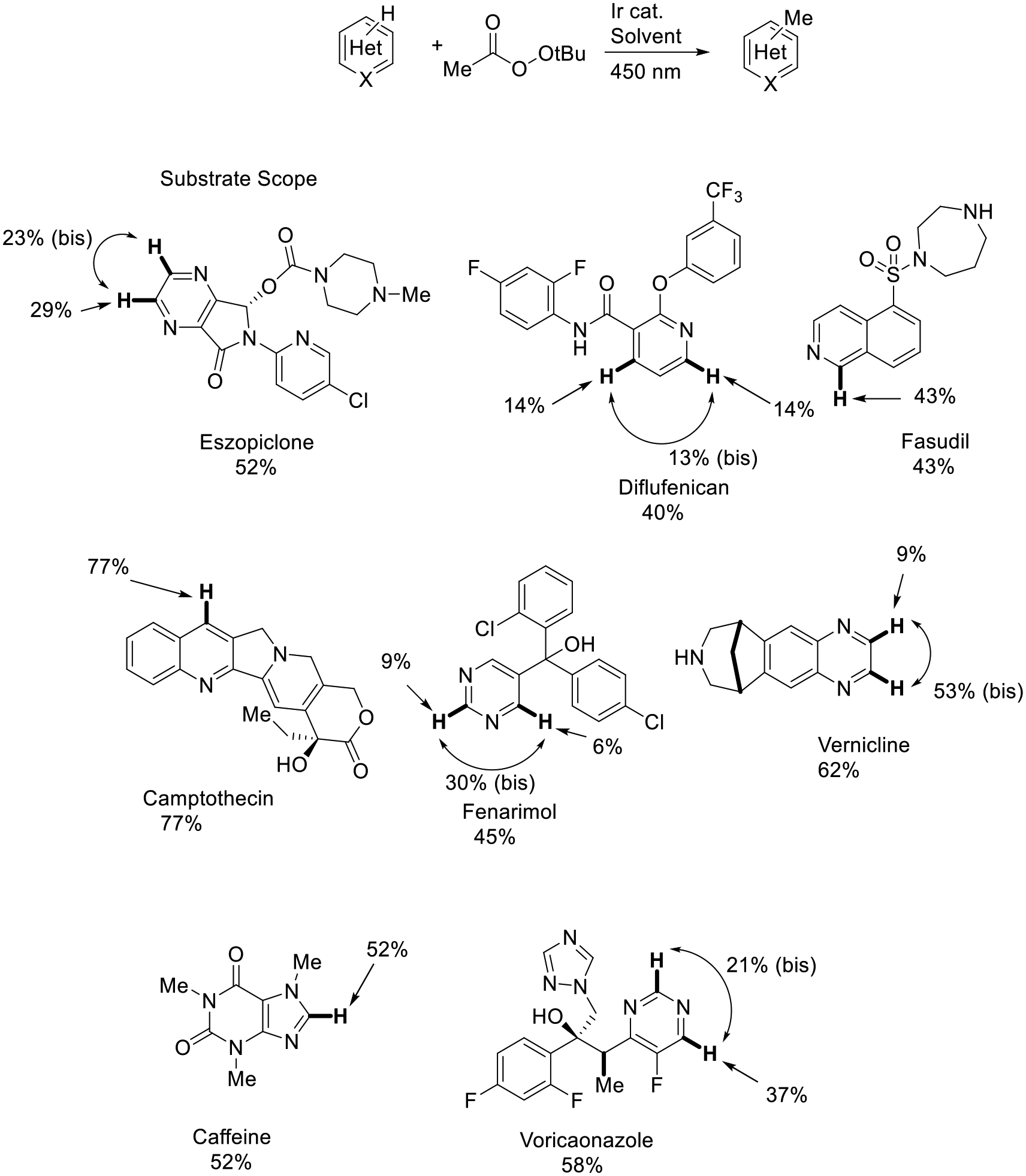
Late-stage functionalization of complex heterocycles.

In the year 2014, DiRocco and colleagues described a late-stage functionalization of heterocycles strategy through photoredox catalysis. The tert-Butoxy radical (*t*-BuO•) was produced by using an Ir^III^ catalyst, 450 nm light, and trifluoracetic acid (TFA). The key species in the transformation, methyl radical, is achieved through β-scission of the *t*-BuO•. The addition of the methyl radical to heterocycles realized the direct C_sp2_−Hydrogen alkylation of heteroarenes. This unique reaction condition was used to activate bioactive molecules, including eszopiclone, diflufenican, fasudil, camptothecin, fenarimol, vernicline, caffeine, and voriconazole in moderate yields (40–77%, Scheme 2) [50]. The method would potentially expand the late-stage functionalization toolbox for medicinal chemists.

**Scheme 3.**
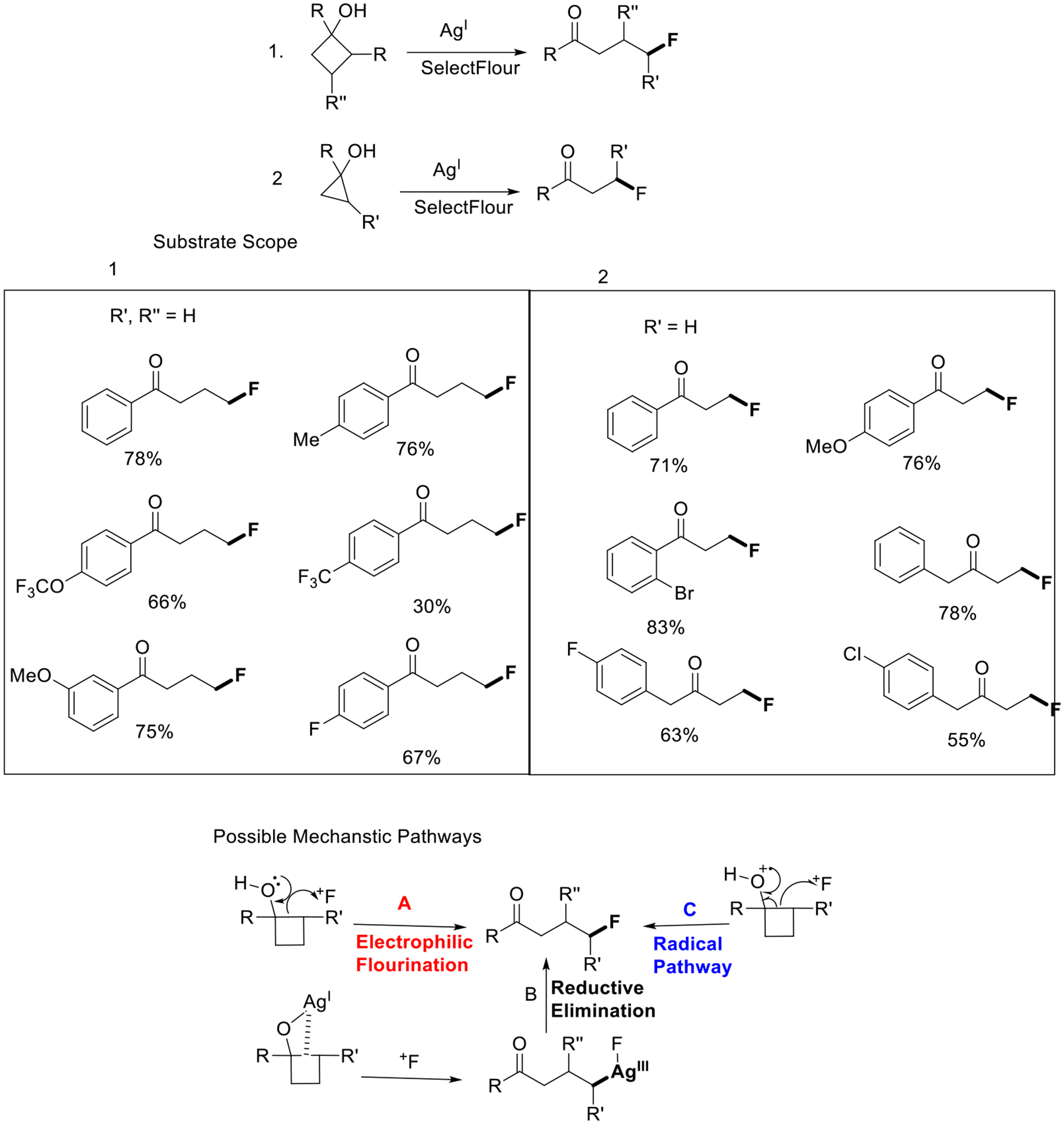
Synthesis of ɣ- and β-fluorinated ketones and possible mechanistic pathways.

The utilization of fluorinated ketones presents a convenient and effective means of introducing fluorine atoms into intricate molecular structures. While the incorporation of α-fluorinated ketones is relatively straightforward, achieved by converting the acidic α-hydrogen of the ketone through various electrophilic fluorinating agents, the direct fluorination process for synthesizing fluorinated ketones remains infrequently documented. However, a notable breakthrough has been made by Zhu and colleagues, who devised an efficient methodology for producing β- and γ-fluorinated ketones. The Ag-catalyzed ring-opening approach involves a sequential sequence of bond cleavage and formation reactions, enabling the generation of a broad range of substrates with favorable regioselectivity. In relation to the potential mechanism, three pathways may be involved. Firstly, when the electrophilic fluorinating species is present, the four-membered ring can potentially undergo direct opening without requiring the Ag catalyst (Path A). Another possibility is the Ag-assisted ring-opening process (Path B). Similar to the Rh^I^-catalyzed ring-opening reactions, Ag^I^ coordinates with the hydroxyl group and inserts itself into the neighboring C−C bond, resulting in the formation of a C−Ag bond. After reacting with SelectFluor, the resulting Ag^III^ intermediate endures reductive elimination, leading to the formation of the desired product. The third pathway involves a radical mechanism (Path C) (Scheme 3) [51].

**Scheme 4.**
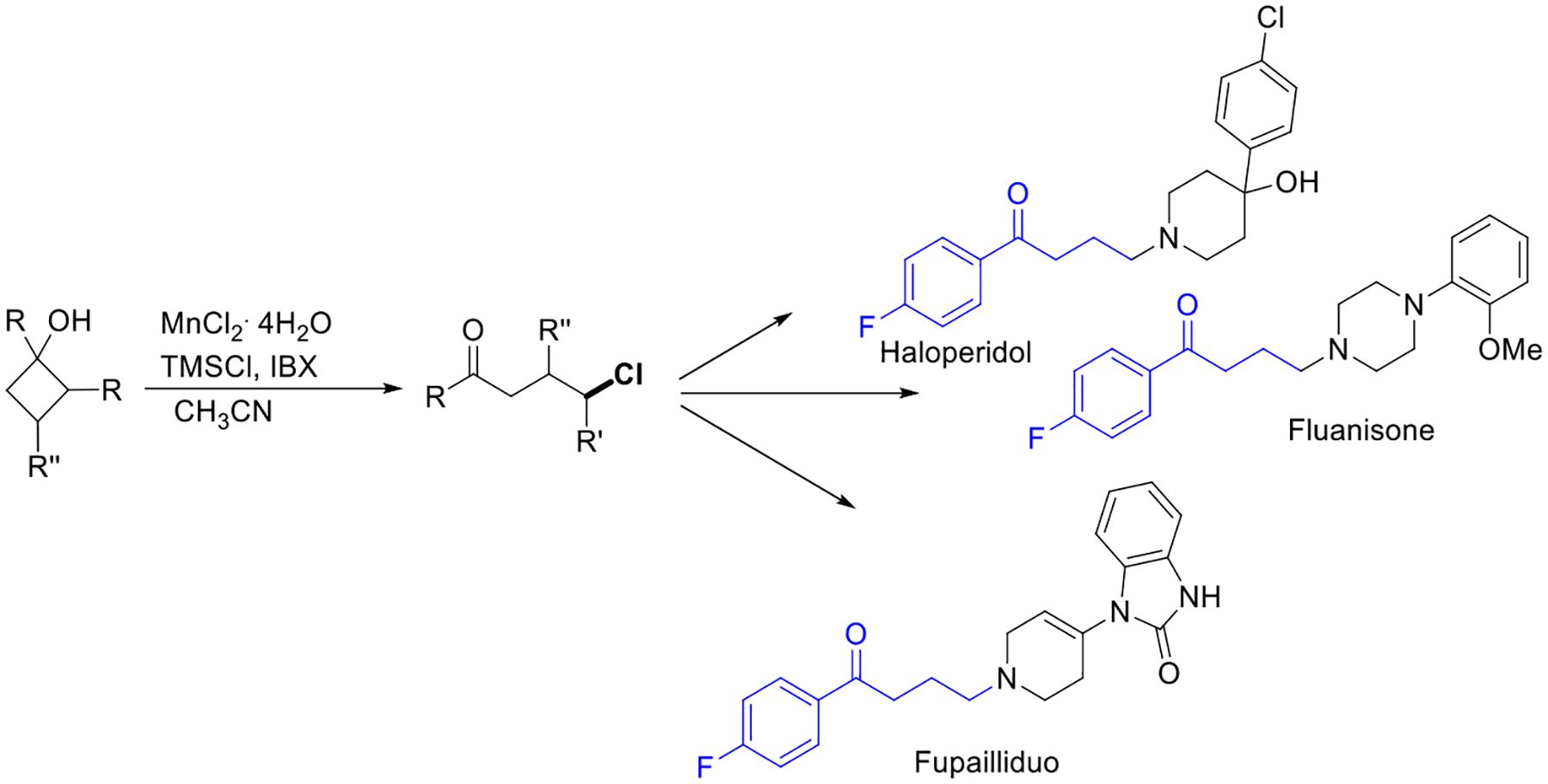
Ring-opening transformation for preparation of bioactive molecules.

In 2016, Chen’s group reported another work on an effective protocol for the ring-opening chlorination process of cyclobutanols via alkoxy radical formation using a manganese catalyst. These mild reaction conditions were applied to various functionalities of acyclic ketones and cyclic chlorides and achieved excellent product yield. The efficient method could lead to an important intermediate, which is utilized for direct access to biologically active compounds such as Haloperidol, Fluanisone, and Fupailliduo (Scheme 4) [52].

**Scheme 5.**
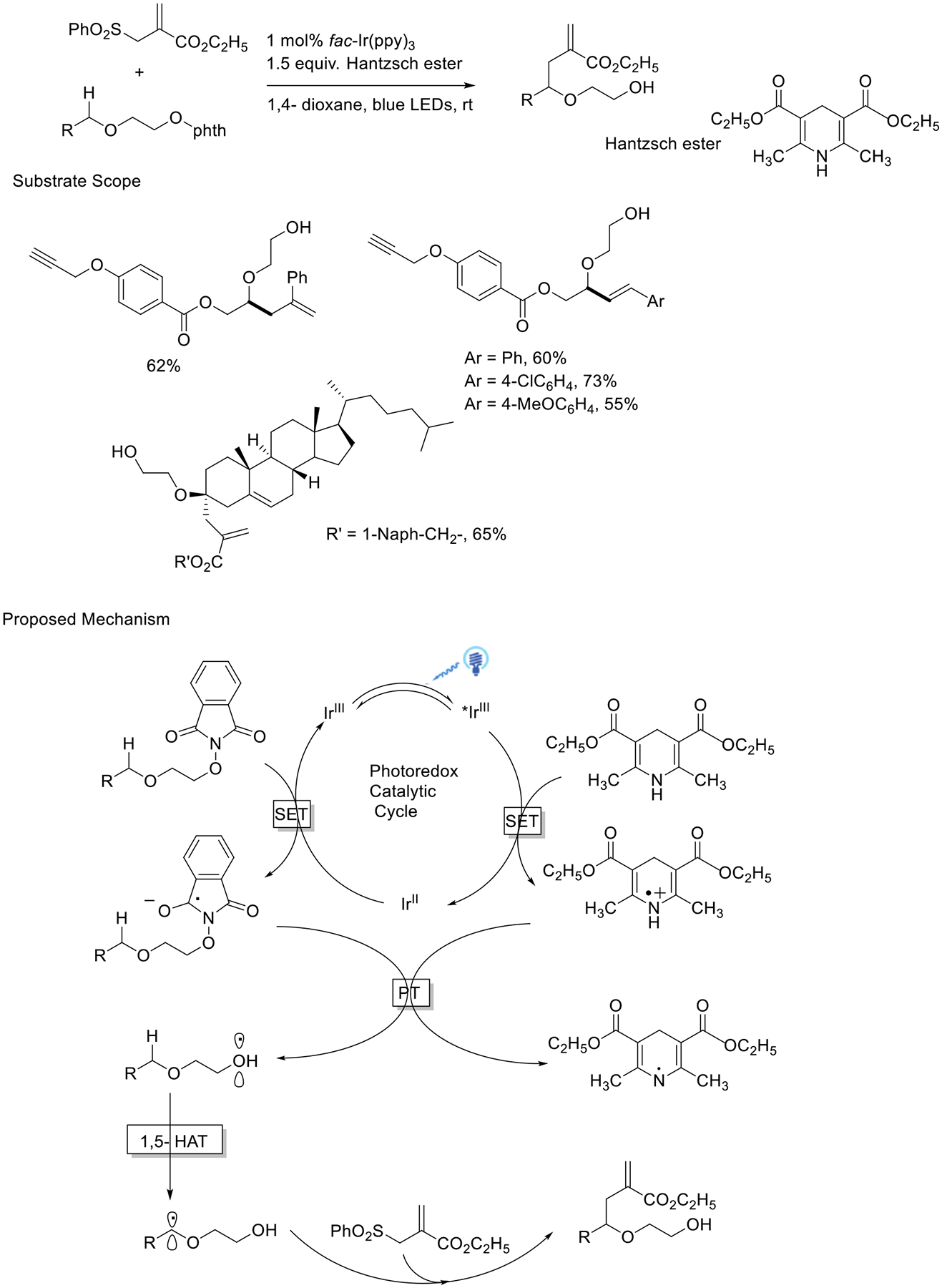
Alkoxyl radical enabled C_sp3_−H functionalization.

Chen and coworkers described a process for the photocatalytic-promoted generation of oxygen-centered radicals using *N*-alkoxy-phthalimide as a radical precursor. The authors performed luminescence quenching experiments and showed that the Hantzsch ester demonstrates efficient quenching of the photoexcited fac-[Ir(ppy)3]*, indicating that the predominant reaction pathway for generating the Ir^II^ intermediate is through reductive quenching. This protocol was applied to achieve selective allylation and alkenylation on activated and inactivated Csp^3^-H bonds. Furthermore, this alkoxyl radical system was utilized in the functionalization of complex molecules or biomolecules (Scheme 5) [53].

**Scheme 6.**
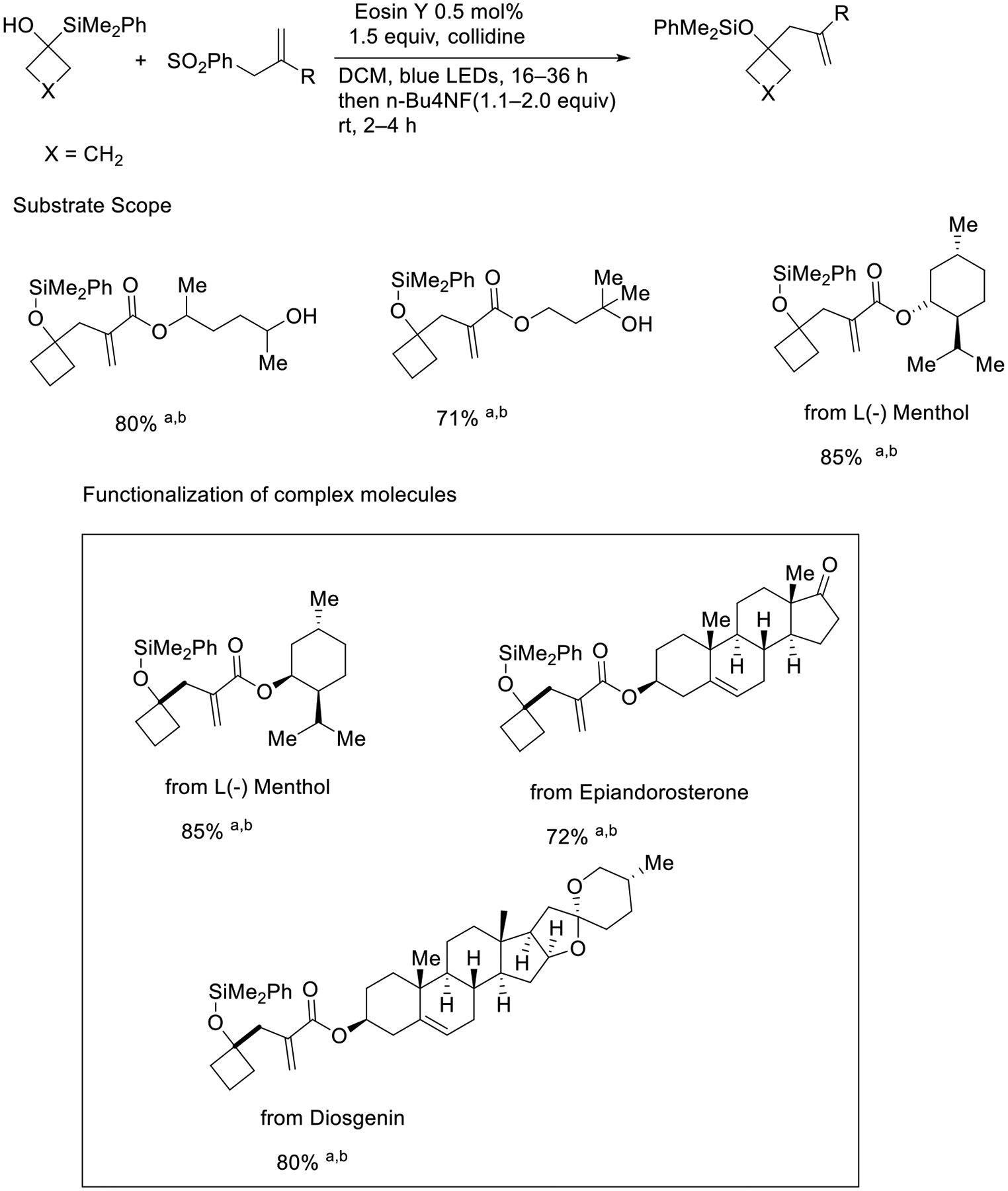
Allylic sulfone activation via 1,2-silyl transfer. a = [Ir(dF(CF3)ppy)2(5,50-d(CF3)bpy)]PF6 (2 mol %) was used as the catalyst instead of Eosin Y. b = No desilylation step.

In the year 2021, Xiao Shen and coworkers introduced 1,2-silyl transfer to functionalized allylic sulfones. The key factor of this approach is that when the silyl group attaches to the α-position of alcohol, it can avoid β-carbon elimination. This synthetic approach was used for the synthesis of L(−) Menthol, Epiandrosterone, and Diosgenin analogs in great yields (72–85%, Scheme 6) [54].

**Scheme 7.**
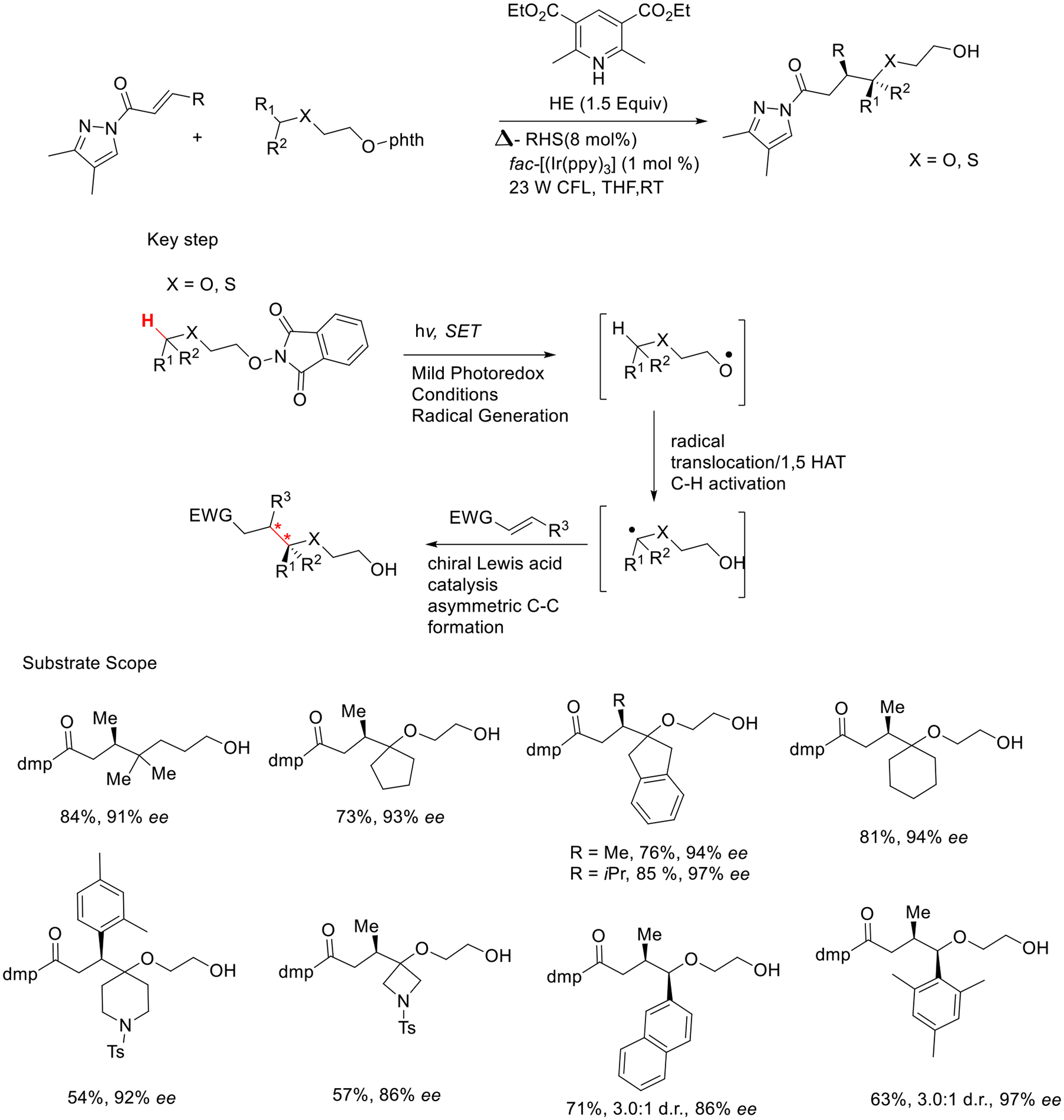
Photoredox catalytic C-H activation via radical approach.

Moreover, Meggers’s research group presented a combination of photoredox systems with chiral Lewis acid catalysis for C-H activation through alkoxy radical chemistry. This protocol shows significant tolerance on a wide array of functionalities of C-H bonds and simultaneously introduces two stereocenters. This approach utilizes a photoredox-mediated transformation of an alkoxy phthalimide compound into an alkoxy radical that subsequently converts to a carbon radical through 1,5-HAT. The resulting carbon radical then undergoes an intermolecular addition to an alkene species. It uses recently discovered redox-active radical intermediate, namely N-alkoxyphthalimides and N-acylpyrazoles, as new acidic functionality (Scheme 7) [55]. The method provided not only good-to-great yields (54–86%) but also good-to-excellent enantioselectivity (86% ee–97% ee).

**Scheme 8.**
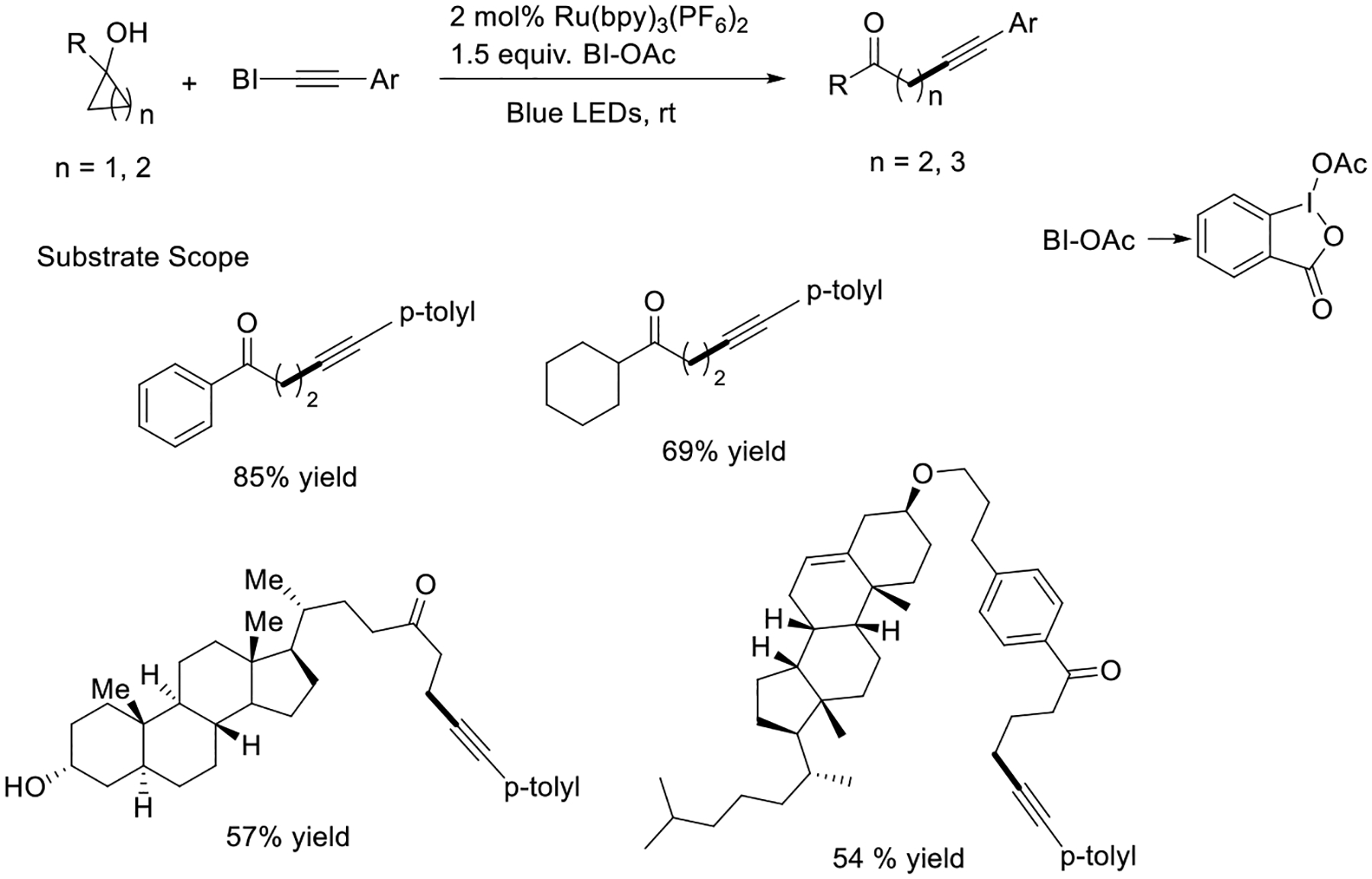
Visible light-promoted carbon–carbon bond cleavage and functionalization.

In the same year, Chen et al.’s other report showed a ruthenium photocatalytic system leads to the formation of structurally complex steroidals using visible light energy. Both alkenyl and alkynyl functionality were introduced in strained cycloalkanol with the assistance of benziodoxole reagents. In this report, visible light-induced alcohol oxidation generated alkoxy radical species via cyclic iodine intermediate (Scheme 8) [56].

**Scheme 9.**
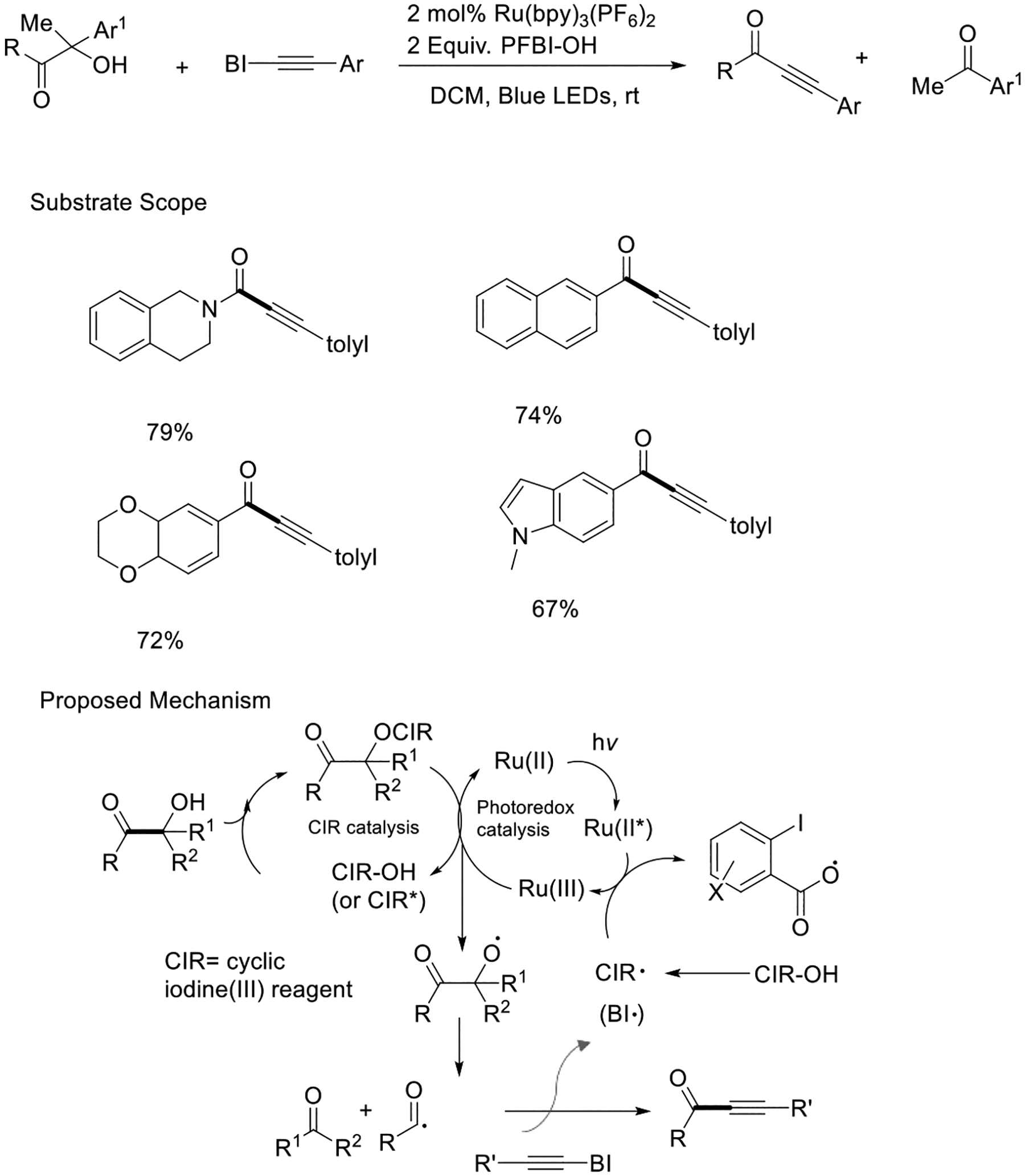
Visible light-mediated alkynylation of β-carbonyl alcohols.

In 2017, Chen and coworkers described the selective carbonyl-C(sp^3^) bond scission in various functional groups such as amides, esters, and alcohols in a regio- and chemoselective manner under modest conditions using a ruthenium photocatalyst. The reaction benefits from the catalytic properties of the cyclic iodine-III reagent, affording model compounds effectively. These findings support the hypothesis that the complex of β-carbonyl alcohol and CIR works as a crucial intermediate in the process. Within the photocatalytic system, this intermediate undergoes oxidation, leading to the β-carbonyl alkoxyl radical. In the photocatalytic process, it is hypothesized that the Ru(bpy)_3_^2+^* species was obtained upon light irradiation and was subsequently oxidized to Ru(bpy)_3_^3+^. Following this step, Ru(bpy)_3_^3+^ oxidizes the in situ formed β-carbonyl alcohol/CIR intermediate, liberating the CIR cation (CIR^+^) and initiating another CIR catalytic cycle. The resulting alkoxyl radical undertakes a bond cleavage reaction at the β-carbonyl-C(sp^3^) position. Such a process can lead to the formation of carbamoyl, alkoxylcarbonyl, or acyl radicals, which subsequently undergo radical addition to the alkynyl benziodoxole. This method is applied for the synthesis of complex synthons such as ynamides, ynoates, and ynones (Scheme 9) [57].

**Scheme 10.**
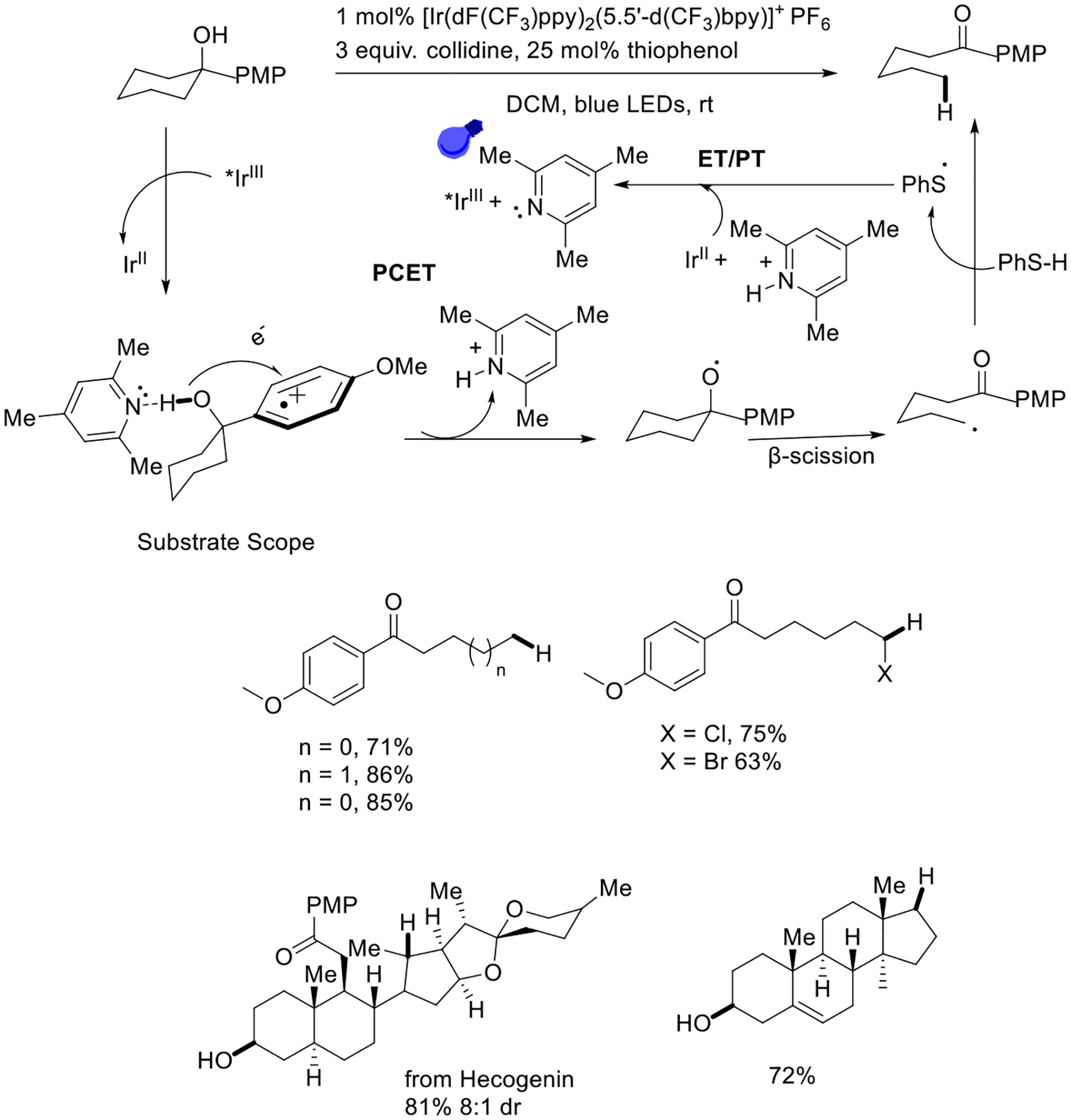
PCET-enabled activation on unstrained cyclic alcohols. PMP = *para*-methoxyphenyl.

Meanwhile, the Knowles group has successfully developed a unique ring-opening protocol that proceeds via β-scission, applying an iridium photocatalyst induced by a visible light source. This protocol achieved isomerization of cyclic alcohol that led to the synthesis of a hecogenin analog. Mechanistic studies reveal that the proton-coupled electron transfer approach triggered O-H bond homolysis and delivered a oxygen-centered radical. It is proposed that the radical cation species attracted the electron in coordination with a proton moves to a weak base (Scheme 10) [58].

**Scheme 11.**
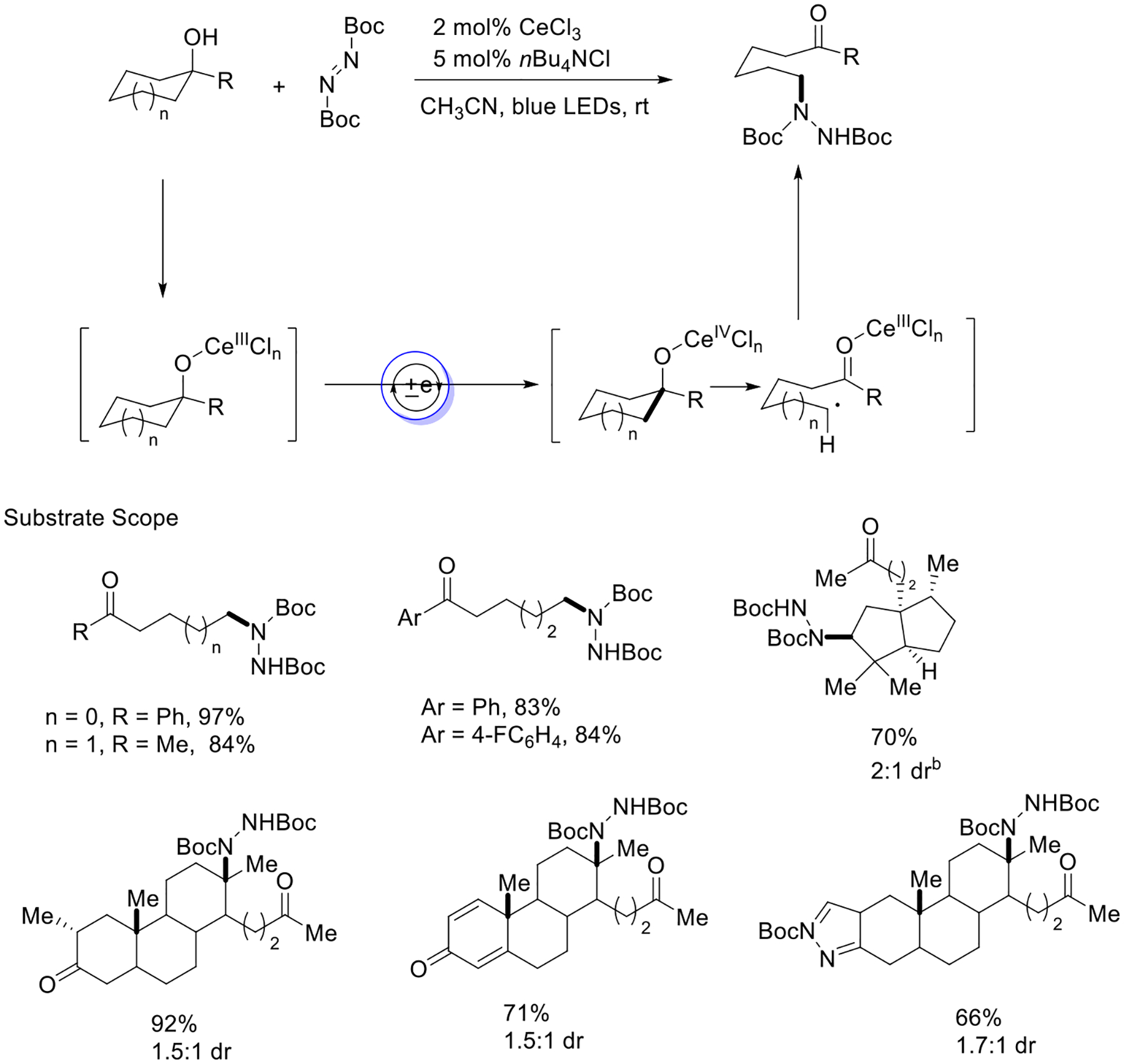
Cerium photocatalyzed reaction of cycloalkanols.

In the same year, Zuo and coworkers published their work based on CeCl_3_-induced alcohol oxidation to generate alkoxyl radicals from unstrained cycloalkanols and achieve amination of the C-C bond of cycloalkanol. This strategy is utilized to rapidly structure complex biomolecules such as Talampanel. Mechanistic investigation disclosed that the cerium alkoxide could be easily photoexcited by visible light and later converted to the corresponding Ce (IV) intermediate. Then, this species could lead to the challenging β-fragmentation and produce an alkyl radical (Scheme 11) [59].

**Scheme 12.**
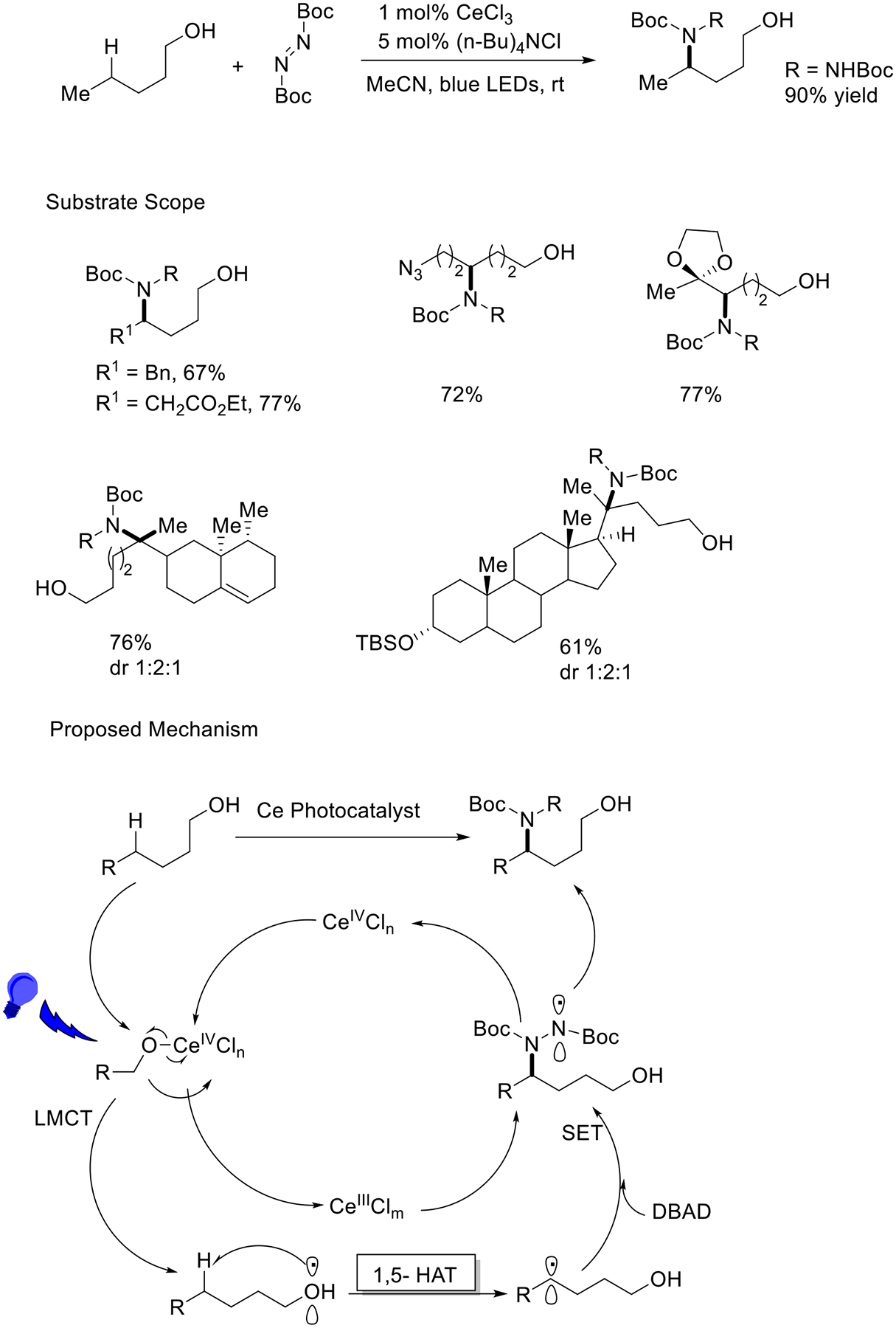
δ selective-C−H functionalization of alcohols.

Achieving control over the specific location and type of C-C bond formation when utilizing unactivated C(sp^3^)-H bonds is a difficult task. However, the use of alkoxyl radicals presents an effective strategy for activating C-H bonds through 1,5-hydrogen atom transfer (1,5-HAT) reactions, mainly due to the higher energy of the O-H bond. When employing different alcohols, such as primary alcohols, in 1,5-HAT reactions, the choice of alcohol can influence both the reactivity and stability of the resulting alkoxy radicals. Primary alcohols generally give rise to more stable alkoxy radicals compared to secondary or tertiary alcohols. This enhanced stability can be attributed to the presence of a single alkyl group attached to the oxygen in primary alcohols, forming a less hindered radical center. The Zuo research group applied a photoinduced LMCT activation mode to activate the free O-H bond in alcohols and produced an alkoxy intermediate using a low-priced cerium photocatalyst via a 1,5 HAT process. This effective catalytic protocol offers selective C-H bond activation using a carbon-linked oxygen radical intermediate. This method delivers novel and simple reaction conditions to achieve a wide array of complex molecules from simple and plentiful alcohols (Scheme 12) [60].

**Scheme 13.**
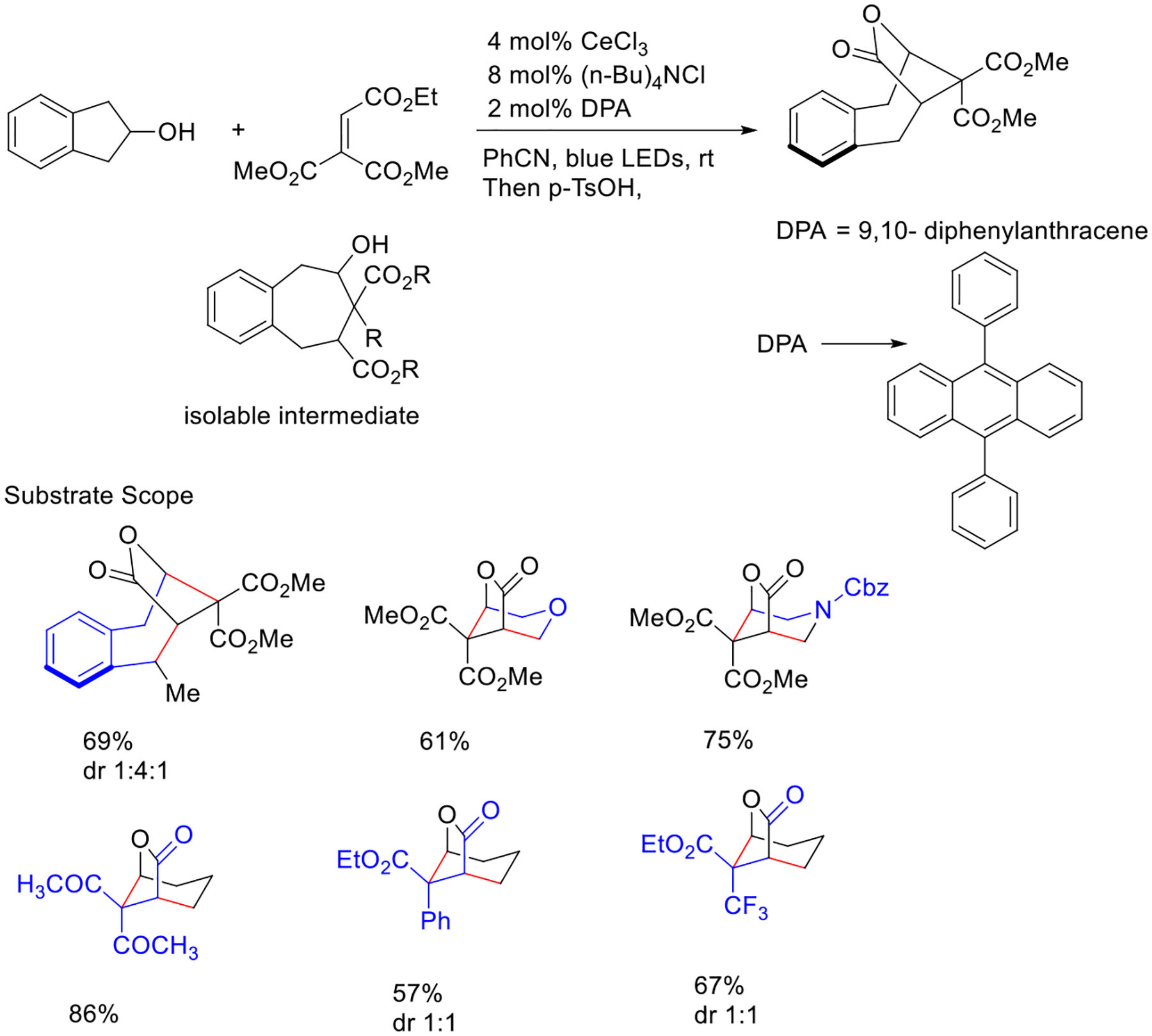
Visible photoredox cerium catalyst promoted β-fragmentation and ring-enlargement of cyclic alcohols for the structure complex bridged lactone.

The same group has described a simple and practical photocatalytic methodology to synthesize bridged lactones through the alkoxy radical approach. Additionally, this protocol was applied for bulk scale synthesis using flow reactors for polycyclic framework. After careful investigation, they hypothesized that upon irradiation, ligand metal charge transfer would lead to homolysis and produce the key carbon-linked oxygen intermediate. After that, this transient radical species initiates a β-fragmentation in cycloalkanol and delivers a carbon radical species. Then, the resultant intermediate is coupled with alkene and creates an additional carbon–carbon bond (Scheme 13) [61].

**Scheme 14.**
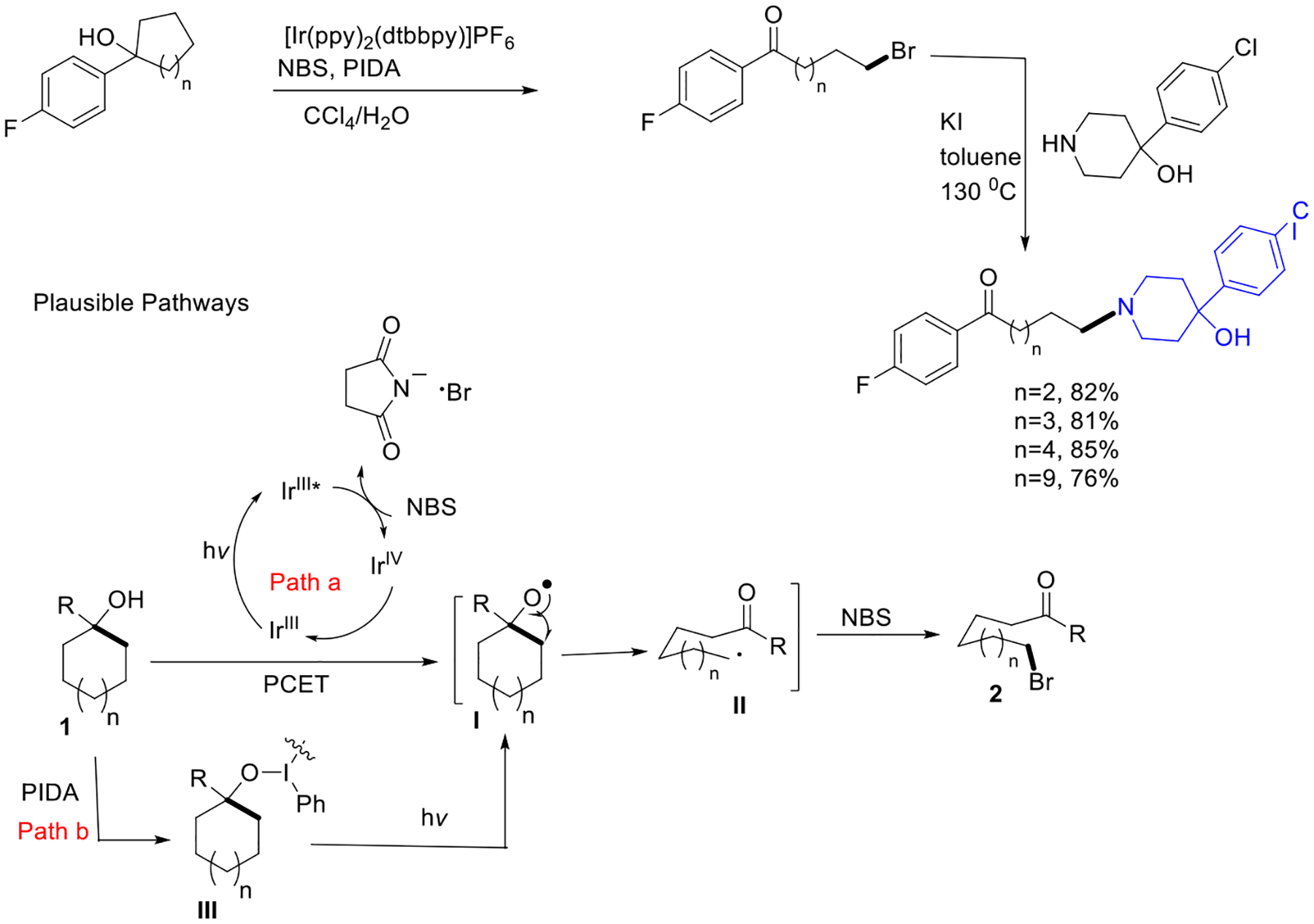
Photoredox ring opening of unstrained cycloalkanols.

During the same period, Zhu’s group presented an advanced photocatalytic ring-opening strategy for various unstrained cyclic alcohols via cyclic C−C cleavage generating alkoxy radicals. This process delivers valuable feedstocks for the production of haloperidol analogs. This group postulated two pathways for alkoxy radical generation. Path a shows that alkoxy radical can be generated via the PCET process employing the Iridium complex and succinimide anion. Alternatively, path b presents that the interaction between cycloalkanol and PIDA leads to homolysis of the O-I bond and produces challenging transient O-radical species (Scheme 14) [62].

**Scheme 15.**
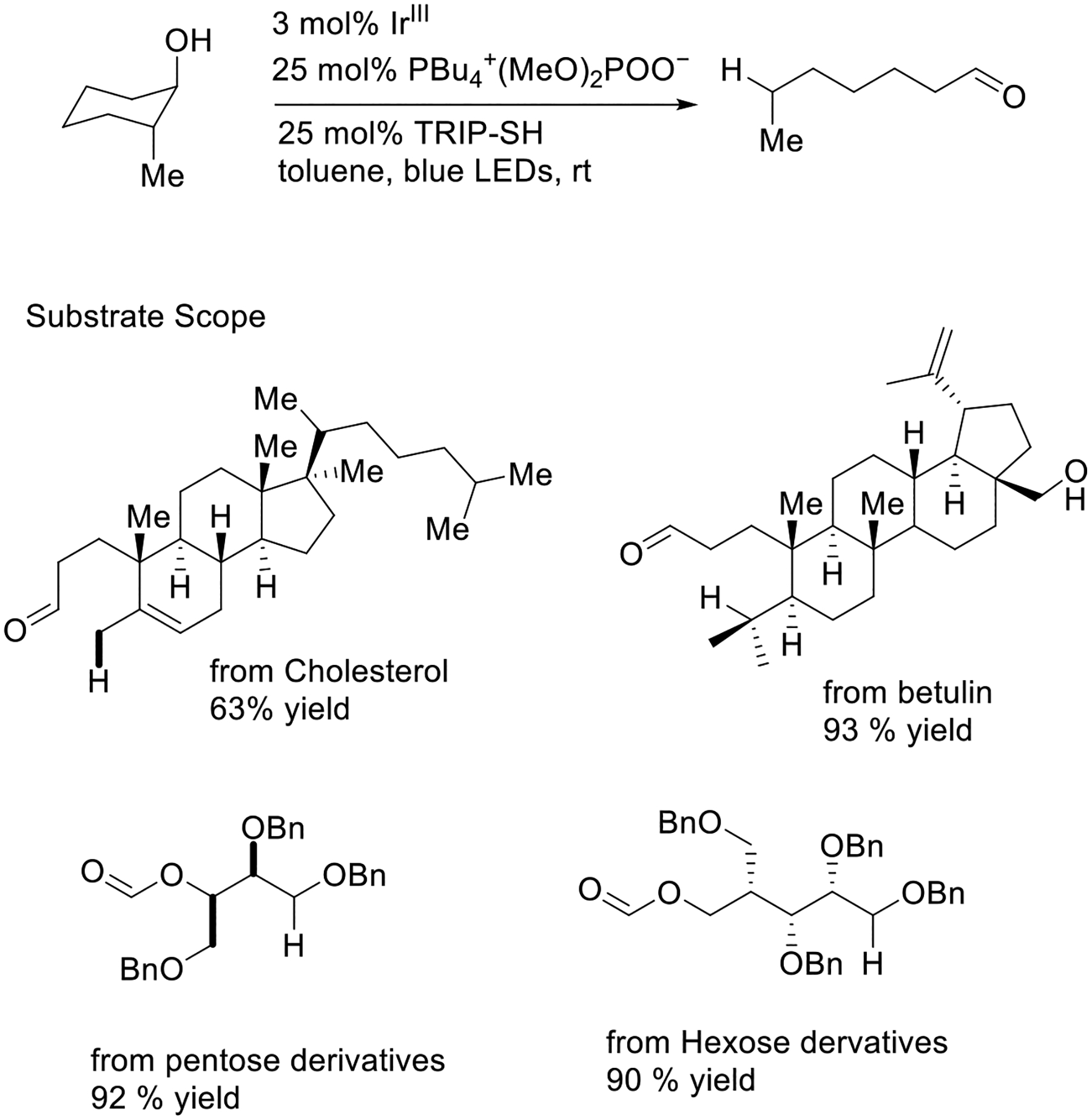
Hydrogenation of complex cyclic alcohol by PCET.

The Knowles group presented the homolytic activation of free alcohol in cycloalkanols via the PCET strategy. This innovative approach could be applied in the hydrogenation of structural derivatives of cholesterol, botulin, pentose, and hexose in great yields (63–93%). This protocol certainly leads as an instance for investigating a new chemical space by activating the small bio-molecules (Scheme 15) [63].

**Scheme 16.**
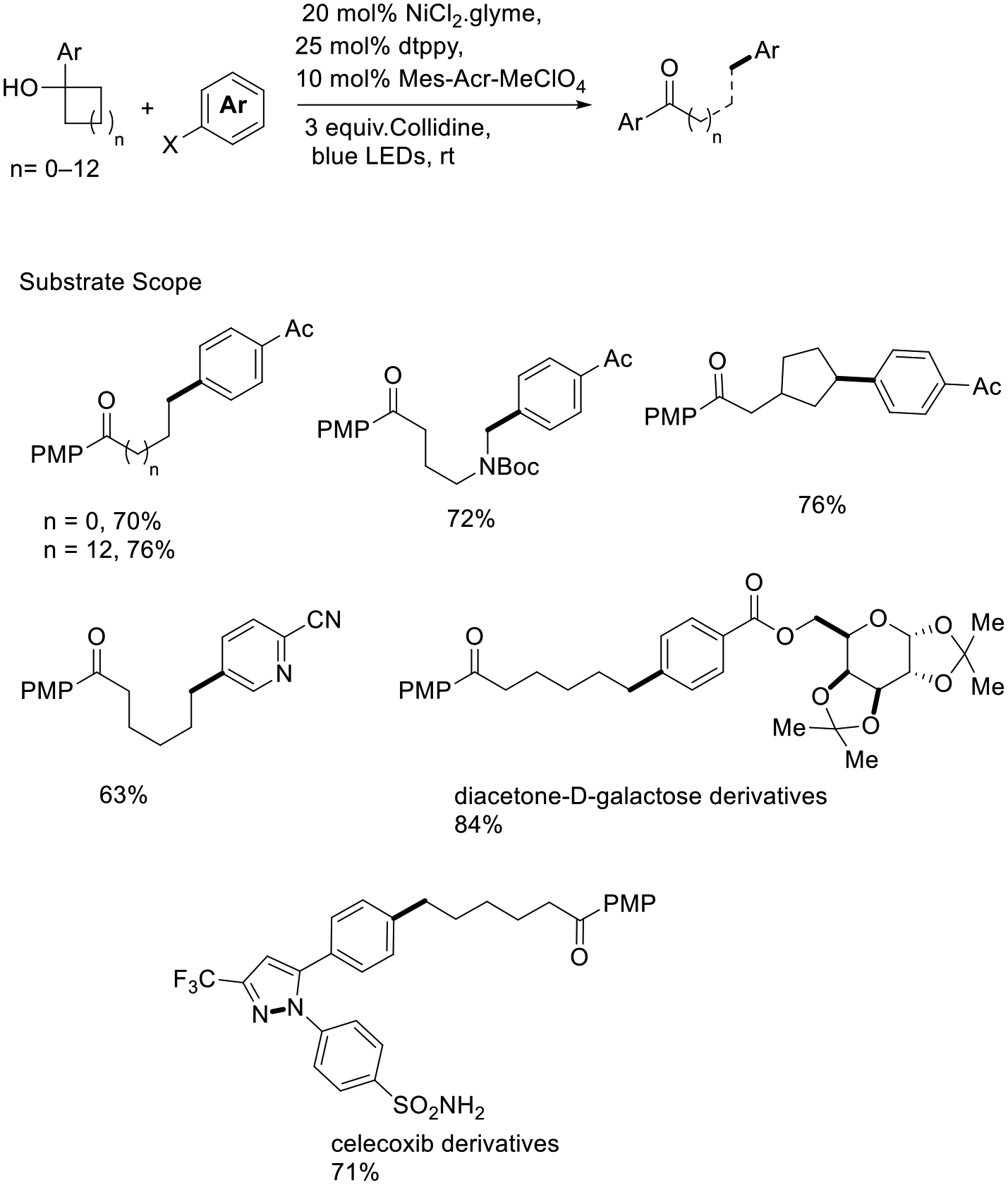
Nickel-catalyzed arylation for late-stage modification.

Rueping and coworkers developed a PCET-enabled nickel photocatalytic methodology for site-specific functionalization of ketones from readily available 3° alcohols by using a strongly oxidizing acridinium salt, which gained high reaction efficiency. This process displays that the lower bond-dissociation free energy of alcohol caused rapid O-radical generation and led to the formation of C-radicals. Then, alkyl radical species were captured by nickel(0), formed a nickel intermediate, and led to the structuring of the provocative C(sp^3^)−C(sp^2^) bond. Thus, a wide range of linear, cyclic, land-bridged alcohols can be successfully explored in this cross-coupling procedure and achieved late-stage modification on Celecoxib and Diacetone-D-galactose derivatives (Scheme 16) [64].

**Scheme 17.**
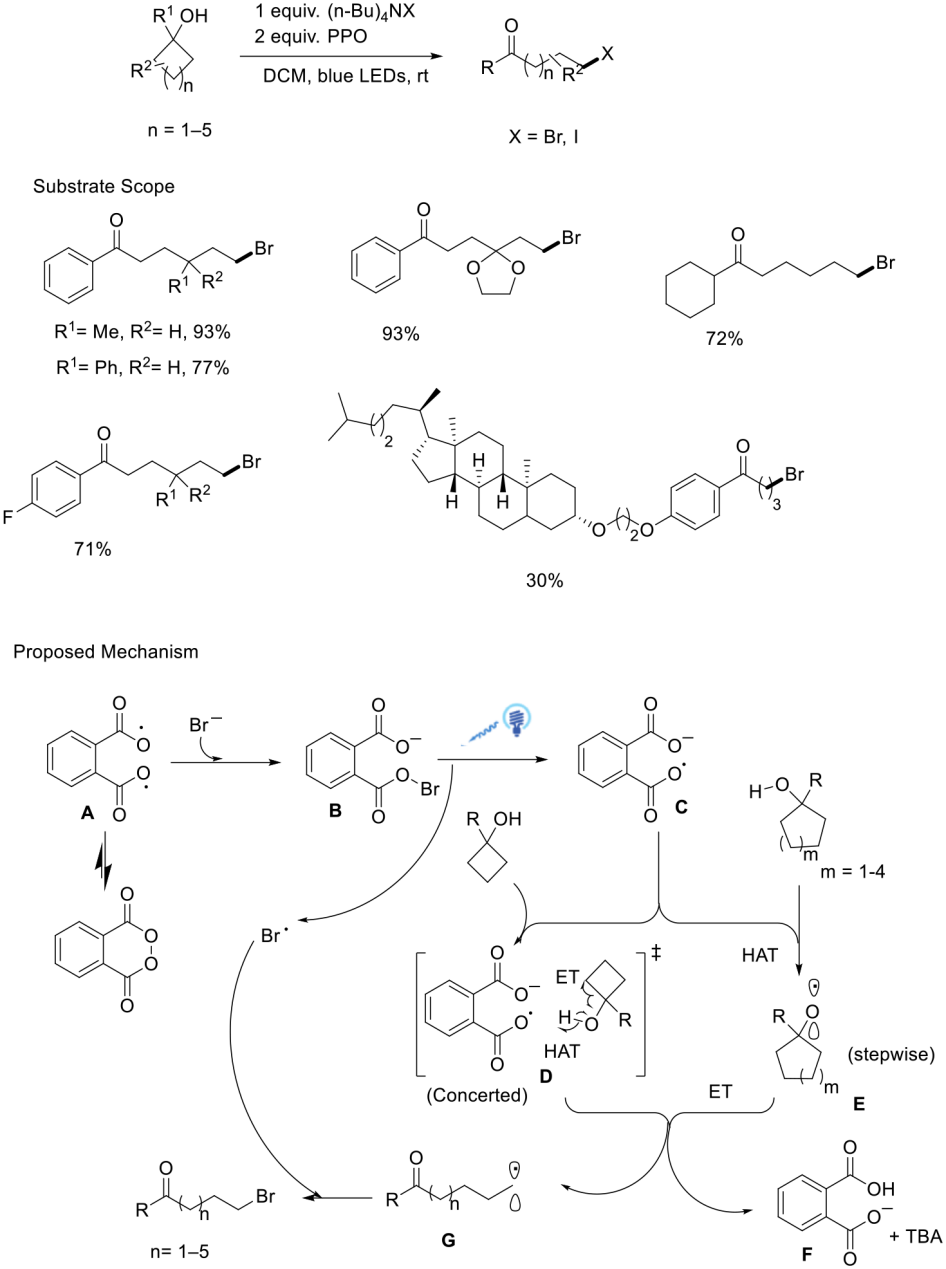
Alkoxy radical induced halogenation of cycloalkanols.

Shi’s group has successfully delivered the halogenation of various cycloalkanols using visible light energy via the ring-opening process. This method used a combination of tetrabutylammonium halide and phthaloyl peroxide under moderate conditions with blue LEDs, achieved a high tolerability for the sensitive functional group, and delivered a wide array of halogenated ketones. This strategy was adopted for the modification of natural product structural units and gained excellent yields. The authors proposed a mechanism according to both experimental results and density functional theory (DFT) calculations. The crucial role of intermediate B, which is generated by the blend of PPO and TBAB, was identified in the step of photolytic O−Br bond cleavage. This cleavage process resulted in the generation of two species: the intermediate **C** and the Br radical. Using cyclobutanol as the substrate, a simultaneous homolysis of the O−H bond and the strained C−C bond occurred. This concerted process directly yielded radical intermediate **G** and the byproduct **F**. On the other hand, cyclic alcohols with five- to eight-membered rings were proposed to go through a stepwise mechanism, initially undergoing hydrogen atom transfer with intermediate **C** to generate alkoxy radical **E**. Finally, carbon radical intermediate **G** reacted with the Br radical, resulting in the formation of the brominated ketones (Scheme 17) [65].

**Scheme 18.**
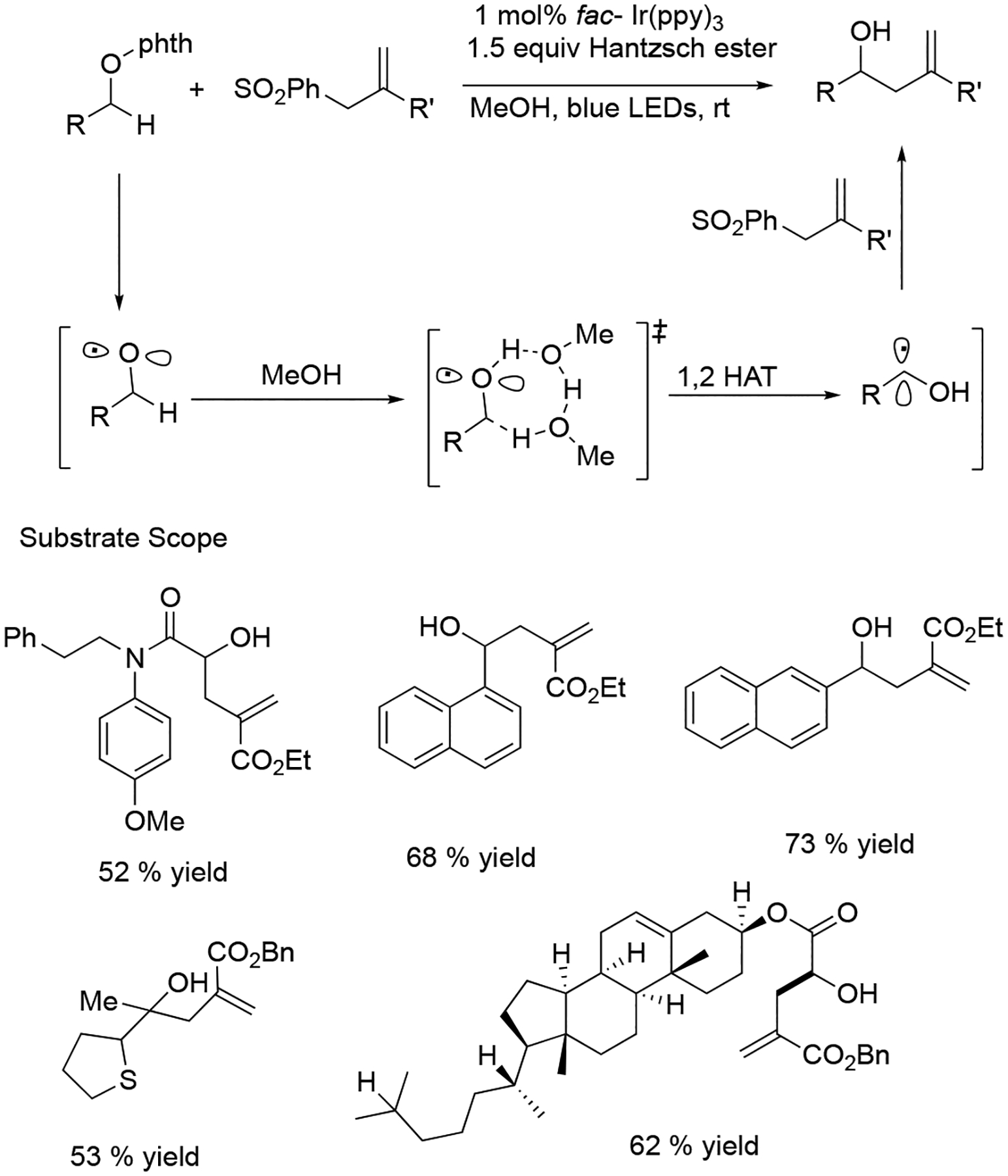
Chemoselective allylation via 1,2-HAT process.

The highly reactive nature of the alkoxyl radicals makes it very useful in organic synthesis. Serving as a reactive intermediate, it allows for the wide range of functionalization of unactivated C-H bonds. When there are δ-C-H bonds in the molecule, the 1,5-HAT reaction is preferred, resulting in the abstraction of the δ-C-H bond. In other cases, when intramolecular δ-C-H bonds are absent, intermolecular HAT reactions become more prominent. It is important to note, however, that intramolecular C-H abstraction by alkoxyl radicals at positions other than the δ-position is less frequently observed because of the presence of unfavorable transition states and high activation energies. So far, there are limited studies on the 1,2-HAT reactivity of alkoxyl radicals, and the practical application of 1,2-HAT for the formation of new C-C bonds is still not well understood or explored. In the year 2020, Chen’s group demonstrated a selective allylation of α-trifluoromethyl, benzylic N-alkoxyl phthalimides, α-carbonyl, and α-cyano using the 1,2-HAT process. The density functional theory calculations, electron paramagnetic resonance results, and mechanistic experiment confirmed that the HAT process is led by the alkoxyl radicals and proved that the activation energy can be minimized by using proton donor solvents in order to accelerate an allylation reaction. This study allowed the rapid synthesis of structurally complex steroid molecules (Scheme 18) [66].

**Scheme 19.**
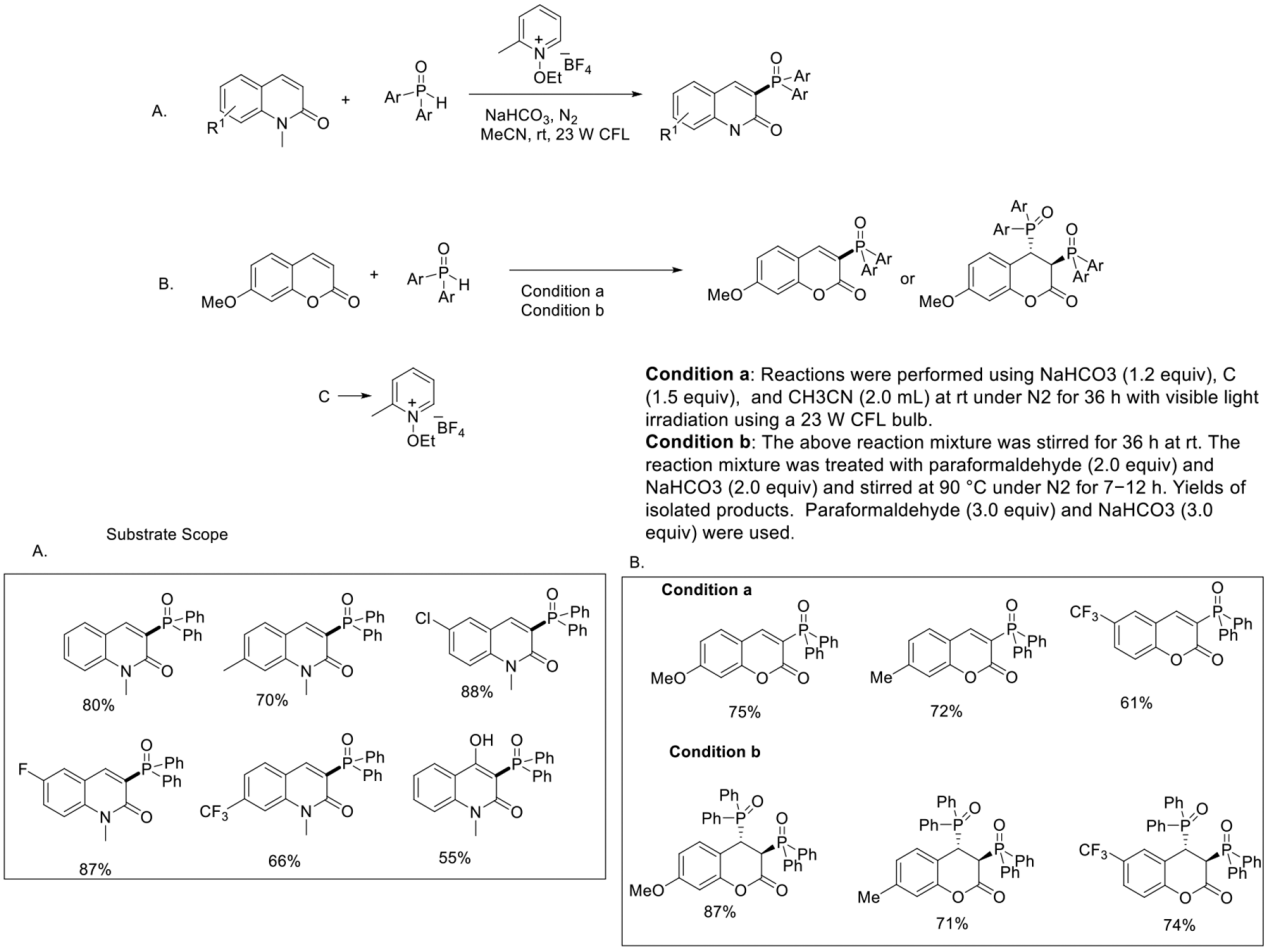
Phosphonation of quinolinones and coumarins.

Shortly after, Hong et al. presented the visible light-enhanced phosphorylation of natural analogs such as coumarins and quinolinones through an alkoxy intermediate-promoted intermolecular hydrogen atom transfer process. They used diphenyl phosphine as a phosphor donor functionality in this reaction. In this methodology, the N-alkoxy-pyridinium precursor was used as an ethoxy radical donor and worked as the oxidant. This method could be applied for the mono-phosphonation of various functionalized quinolinones in good yields (55–87%, Scheme 19) [67]. This regioselective phosphonation strategy would allow a rapid preparation of 3-phosphonylated derivates, which are an essential structural motif in many bioactive compounds.

**Scheme 20.**
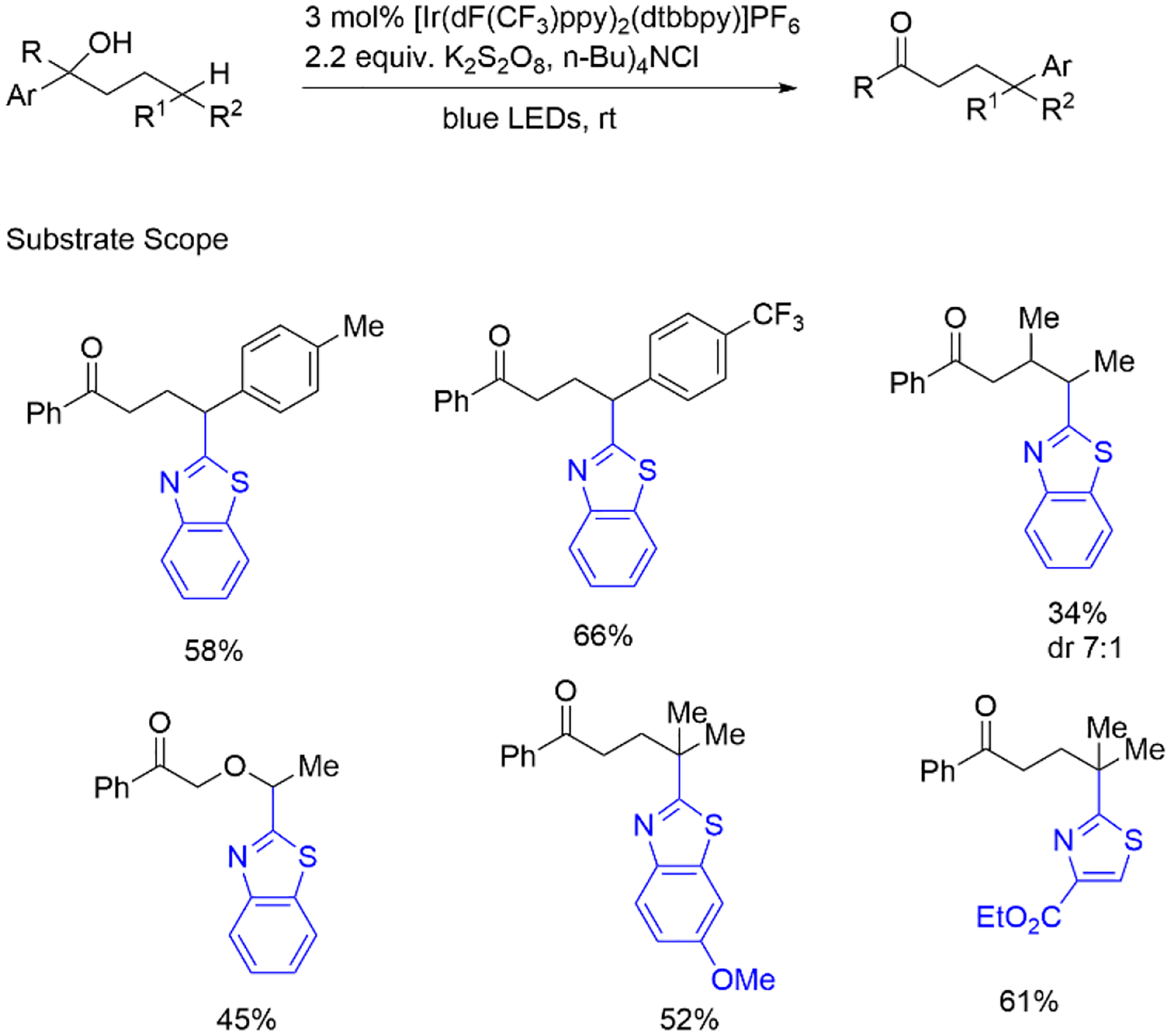
Ir photocatalyst-induced heteroarylation and cyanation.

One year later, Zhu et al. showed alcohol-directed remote C-H activation. The formation of carbon-linked oxygen radical species from the free alcohol O-H bond under a photoredox system leads to successive hydrogen transfer and a heteroaryl moiety. This methodology delivers high tolerability for sensitive functionality to achieve late-stage activation from challenging alcohols (Scheme 20) [68].

**Scheme 21.**
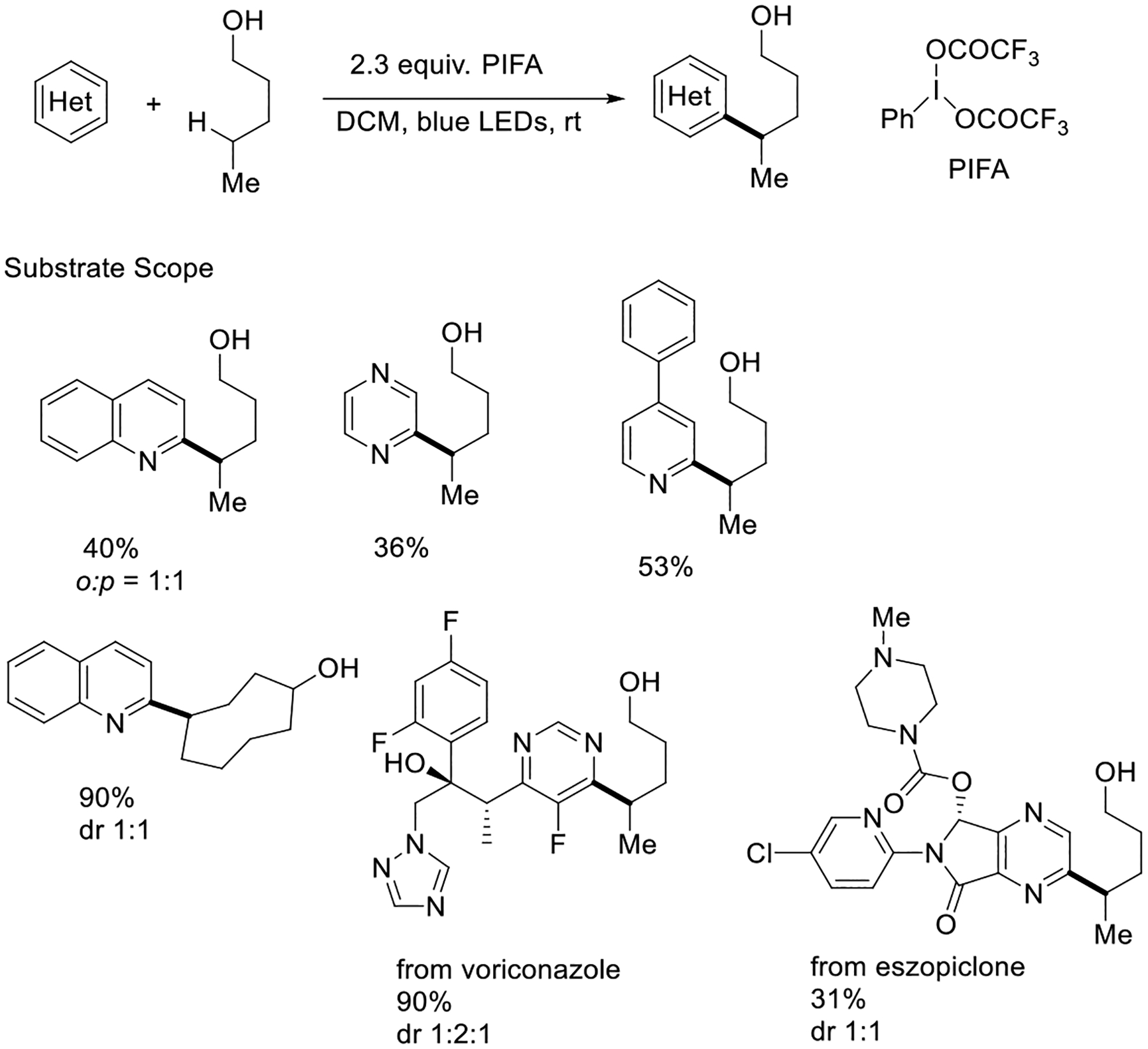
PIFA-mediated photocatalytic generation of alkoxy radicals.

Moreover, the same group developed a facile, economic, and metal-free methodology for non-hypohalite-mediated alkoxy radical generation under 100 W blue LED irradiation conditions, using iodine (III) reagents such as phenyl iodine bis(trifluoroacetate) (PIFA) as the alkoxy radical precursor. This process successfully delivered the Minisci-type products via intermolecular heteroarylation of alcohols. This reaction has displayed that all kinds (1°, 2°, and 3°) of alcohols can generate the alkoxy radical species and can deliver highly regioselective heteroarylation of C(sp3)−H bonds. This protocol structured analogs of voriconazole and eszopiclone (Scheme 21) [69].

**Scheme 22.**
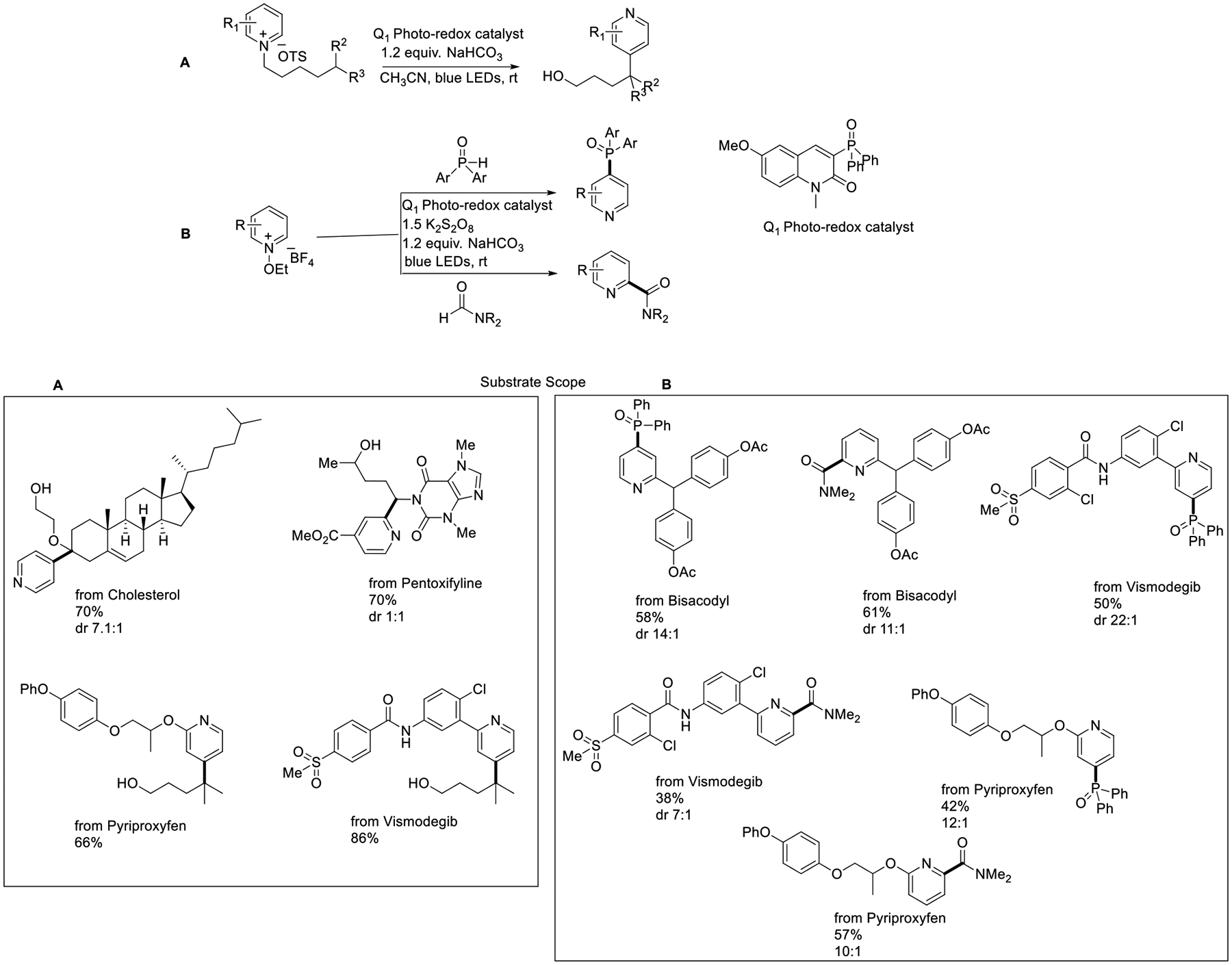
Alkoxy radical mediated Minisic-type reaction and phosphorylation.

In 2018, Hong et al. reported a simple and facile photocatalytic radical distal translocation strategy for Minisic-type heteroarylation and phosphorylation. This method enabled mild reaction conditions, and the quinolinone organic ligand works as a photoredox catalyst. The most important key factor is that the N-alkoxy-pyridinium precursor acts as an alkoxy radical donor and works as a hetero-arylating reagent. This protocol successfully accomplished late-stage modification of cholesterol, pentoxifylline, pyriproxyfen, and vismodegib in yields of 70%, 70%, 66%, and 86%, respectively (Scheme 22A) [70]. Shortly after, a similar strategy was further explored for the functionalization of pyridines, providing a phosphinoylation and carbonylation of pyridines containing bio-active molecules, such as bisacodyl, vismodegib, and pyriproxyfen, in moderate yields (Scheme 22B) [71].

**Scheme 23.**
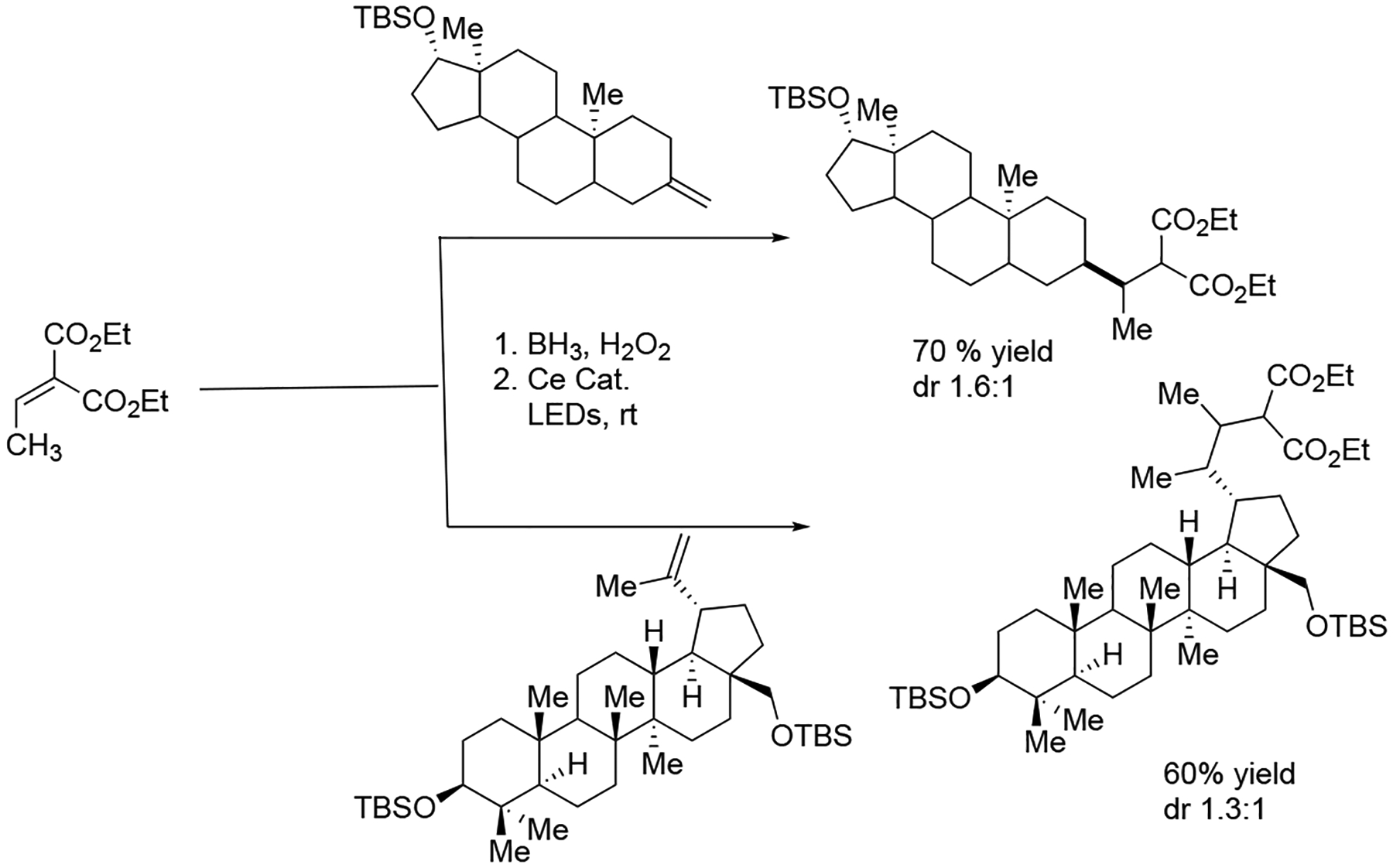
Hydroboration–oxidation and dehydroxymethylation.

Moreover, the Zuo group designed a simple and convenient visible light-induced strategy for the activation of alkanols via radical chemistry. This method effectively delivered hydroboration–oxidation transformations using blue LEDs and low-priced cerium photocatalysts. This operationally facile protocol shows that simple alcohol can lead to the rapid construction of challenging nucleoside structures via an alkoxy radical mechanism (Scheme 23) [72].

**Scheme 24.**
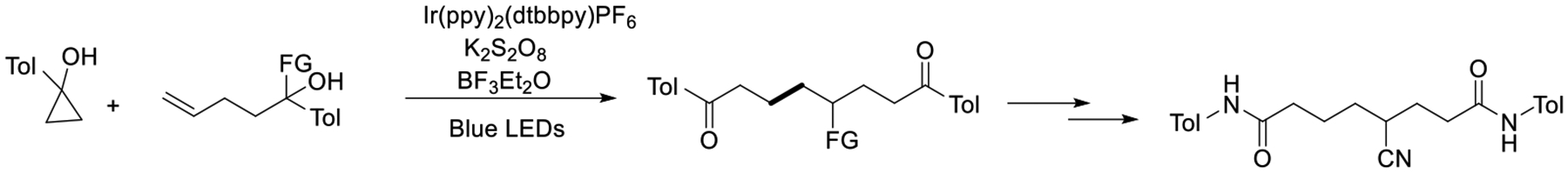
Synthesis of SAHA derivatives.

In addition, Zhu and coworkers reported a successful merging of visible light-induced functional group migration. The reactions were performed under moderate conditions by using an Ir photocatalyst and blue LEDs. Interestingly, BF3OEt2 was used as an additive for promoting the transformation. The reaction provided an unsymmetrical 1,8-dicarbonyl structural motif and utilized the synthesis of SAHA derivatives (Scheme 24) [73].

**Scheme 25.**
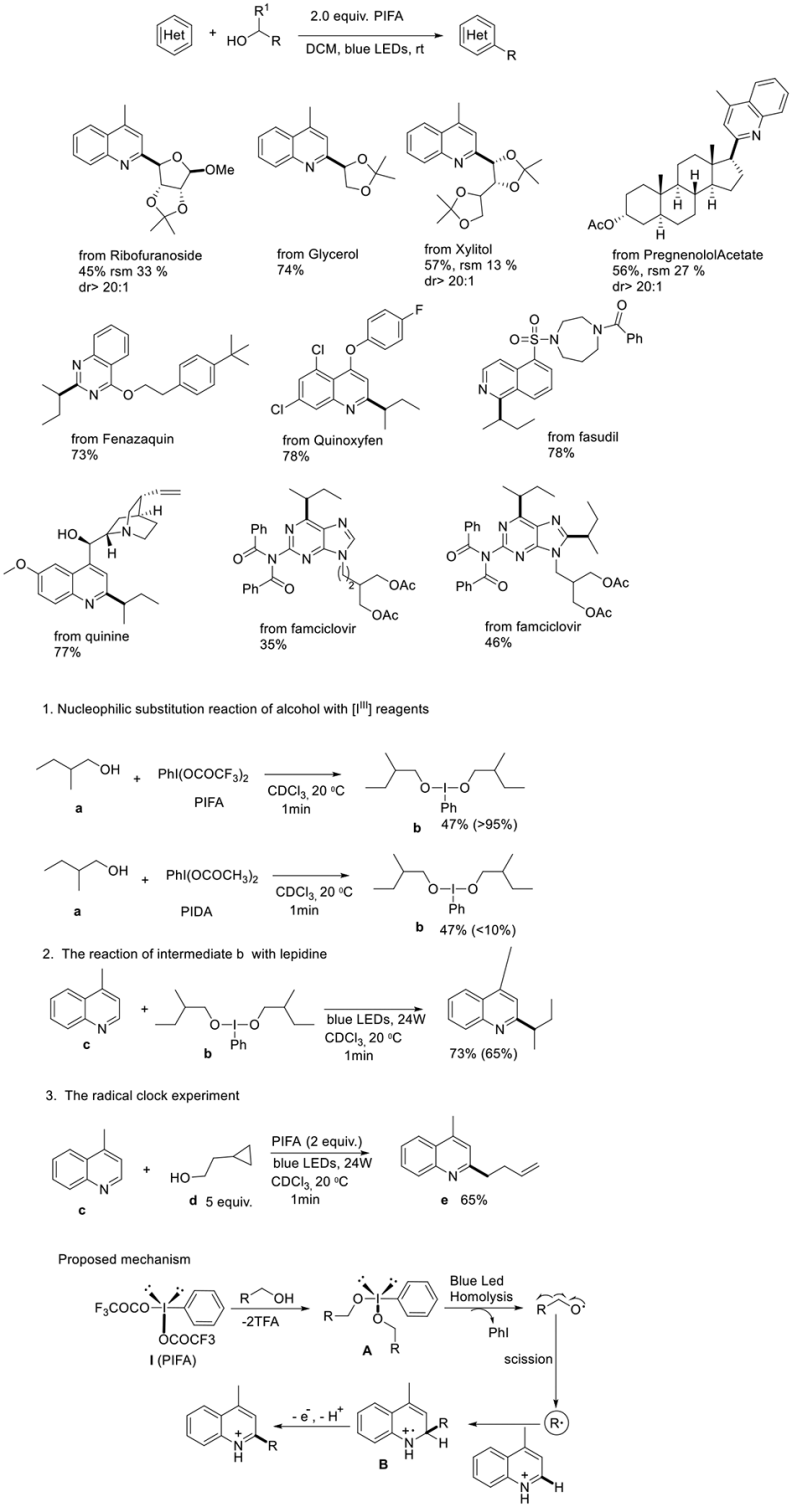
Blue light-induced selective activation in primary alcohols.

The Liu group reported a visible light-induced selective activation in challenging primary alcohols in 2019. This highly selective cleavage was performed under moderate conditions, and [bis(trifluoroacetoxy)iodo]-benzene (PIFA) was used as an oxidant and an activation reagent. Remarkably, a wide variety of natural structural products like sugars and steroid derivatives from ribofuranoside, glycerol, and xylitol were synthesized using this approach. In order to provide a more comprehensive mechanistic understanding, a series of experiments were devised by the author. Initially, the combination of compound (a) and PIFA in CDCl3 promptly yielded a transient intermediate (b), which was deduced based on the analysis of crude 1H-NMR and 13C-NMR. However, when PIDA was reacted with alcohol (a), intermediate (b) was not formed. These findings provide an explanation for the lack of reactivity exhibited by PIDA in cleaving the C(sp3)-C(sp3) bond in alcohols. Subsequently, the reaction of intermediate (b) with lepidine proceeded smoothly, affording the desired product in 73% yield. A radical clock experiment was executed to prove the radical process. The PIFA-promoted reaction between lepidine and 2-cyclopropylethan-1-ol (d) resulted in the ring-opening product (e) in a yield of 65%. These results strongly support the involvement of a free radical pathway, specifically the β-scission of the alkoxyl radical. Based on the experimental results, the authors hypothesized a mechanism and showed that, firstly, PIFA performed nucleophilic substitution with alcohol, resulting in intermediate A. Light-promoted homolytic cleavage of intermediate A delivered a carbon-linked oxygen-centered radical. Subsequently, the alkoxy species experienced β-fragmentation to offer carbon radical species and formaldehyde. Then, the carbon radical reacted with heteroarene and led to the formation of cation B. After that, the radical cation led to single electron oxidation and resulted in the final product (Scheme 25) [74].

**Scheme 26.**
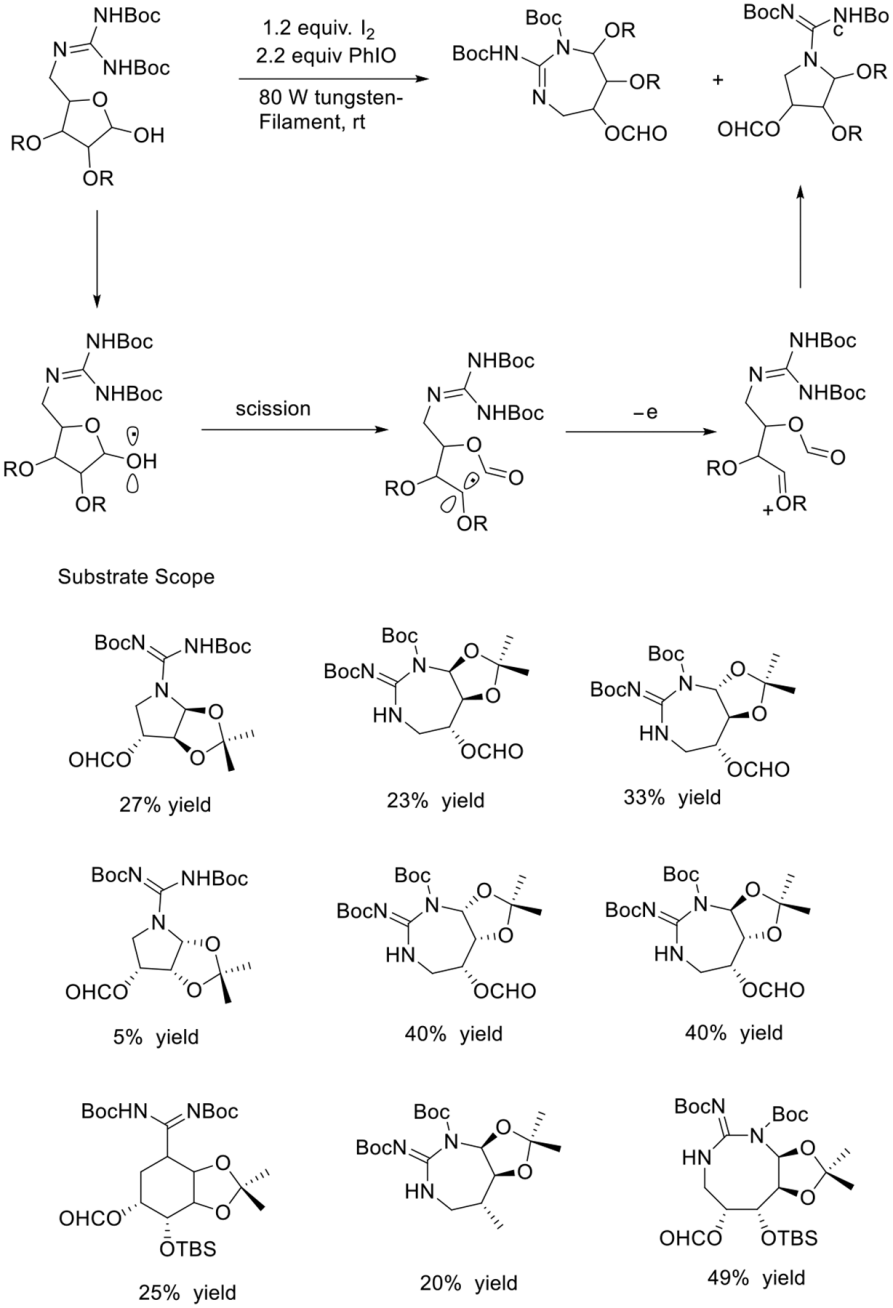
Synthesis of cyclic guanidines

Shortly after, González’s group designed an approach using an organic photocatalyst PhIO and I_2_ combined system and applied it for the synthesis of guanidine derivatives such as (+) Saxitoxin, Crambescin, Tetrodotoxin, and Monanchorin. A key factor in this protocol is that the labile functional group rapidly generates the alkoxy radical intermediate via homolytic scission, which triggers β-fragmentation on the C1–C2 bond and leads to the formation of a new carbon-centered radical intermediate at position C-2 (Scheme 26) [75]. Although the yields is not ideal (5–49%), the method indeed opened a new avenue to the formation of medium-sized guanidine-containing heterocycles.

**Scheme 27.**
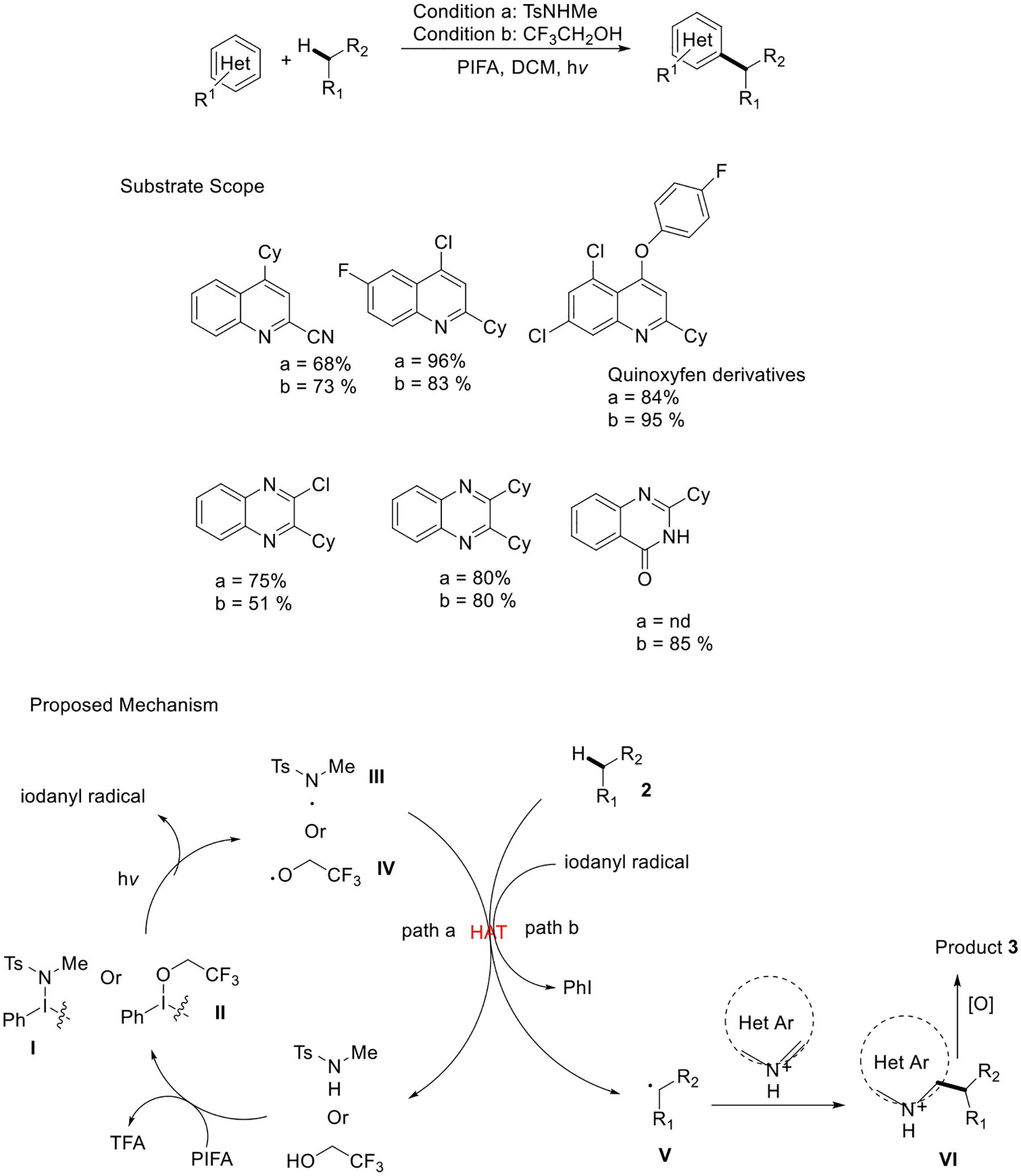
C−H functionalization of simple alkanes in metal-free condition.

The Zhu group reported a metal-free coupling reaction between heteroarenes and simple alkenes via amidyl/alkoxy radical chemistry. This approach delivered mild and neutral reaction conditions and excellent yield for the Minisci reaction products. The interaction between amide/alcohol and PIFA plays a lead role in producing the amidyl/alkoxy radical intermediates. The author presents a proposed mechanism wherein the interaction between a PIFA (PhI(OCOCF_3_)_2_) and an amide or alcohol substrate initiates the formation of intermediate I or II. Upon exposure to visible light, these intermediates undergo homolysis, forming the corresponding amidyl or alkoxy radicals (III or IV). Simultaneously, an iodanyl radical is generated at the same time. The N-radical (or O-radical) then abstracts a hydrogen atom from **2** intermolecularly, leading to C-radical V (path a). Instead, the iodanyl radical can engage in another potential hydrogen atom transfer (HAT) process (path b). It is important to note that the presence of TFA (trifluoroacetic acid) in the reaction mixture activates the N-heteroarene, eliminating the need for additional acid. The addition of alkyl radical intermediate V to the activated heteroarene produces intermediate VI, which would be oxidized to the final product **3**. This novel strategy is applied to the green synthetic process in natural product derivatives (Scheme 27) [76].

**Scheme 28.**
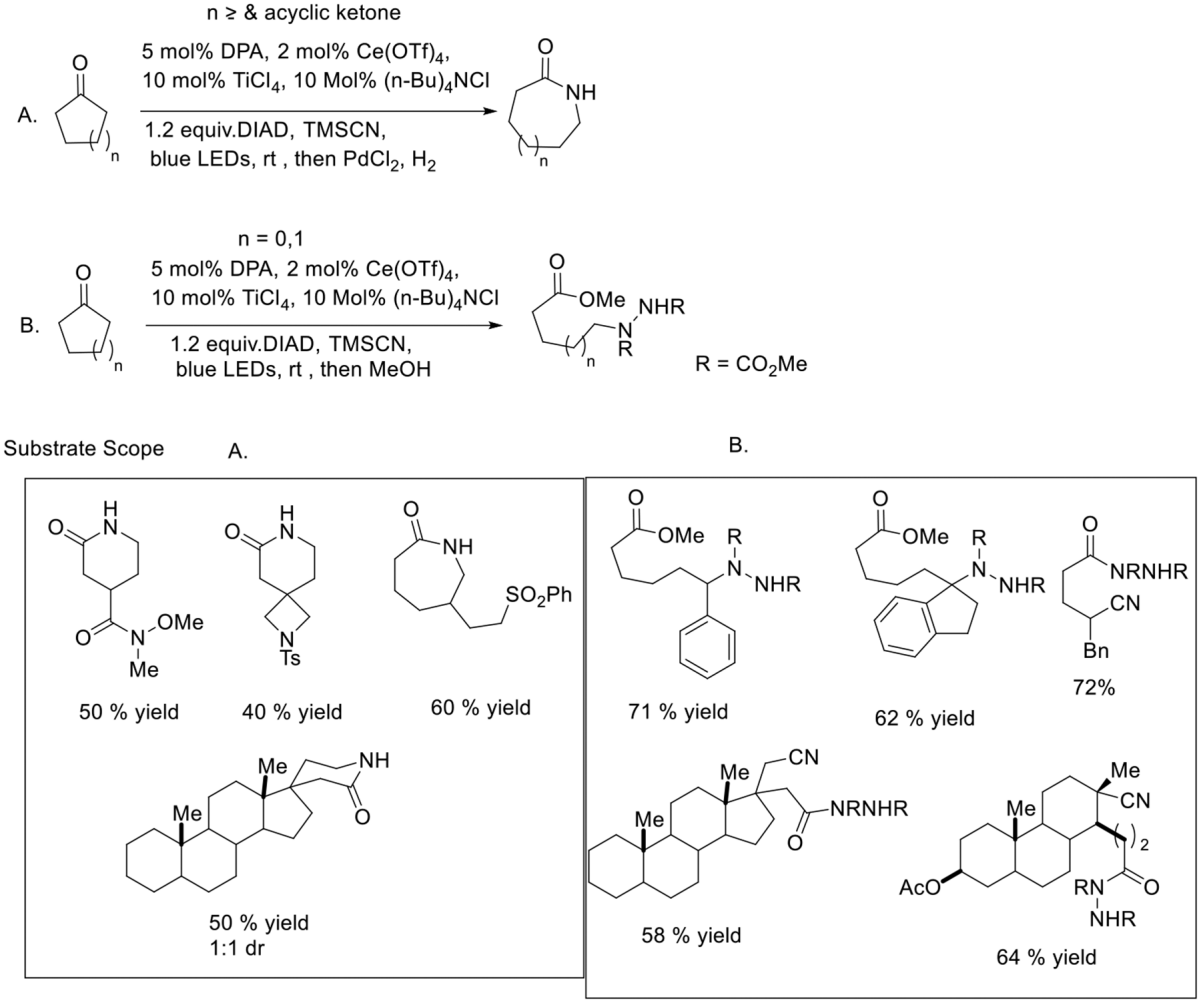
Cerium catalyzed LMCT mode for activation of ketone.

Zuo and coworkers have developed a practical and comprehensive strategy for selectively breaking and modifying C-C bonds within ketones. They have successfully harnessed the ligand-to-metal charge transfer (LMCT) excitation mode to specifically cleave C-C bonds in ketones while simultaneously introducing diverse functional groups to the substrates. This process is carried out under straightforward conditions, utilizing cost-effective cerium catalysts and simple blue LED light. Remarkably, this method demonstrates broad applicability to a wide range of substrates. Both cyclic ketones and acyclic ones work well under this condition. In addition, the reaction condition could be applied to not only simple ketones but also complex functionalized androsterone, thereby enabling their transformation into versatile chemical building blocks. It is noteworthy that this photocatalytic process achieves exceptional regioselectivity in all cases. Such an innovative photocatalytic approach offers a great alternative to the Norrish type I reaction with more selectivity, paving the way to exciting opportunities in more C-C bond cleavage synthetic strategies (Scheme 28) [77].

**Scheme 29.**
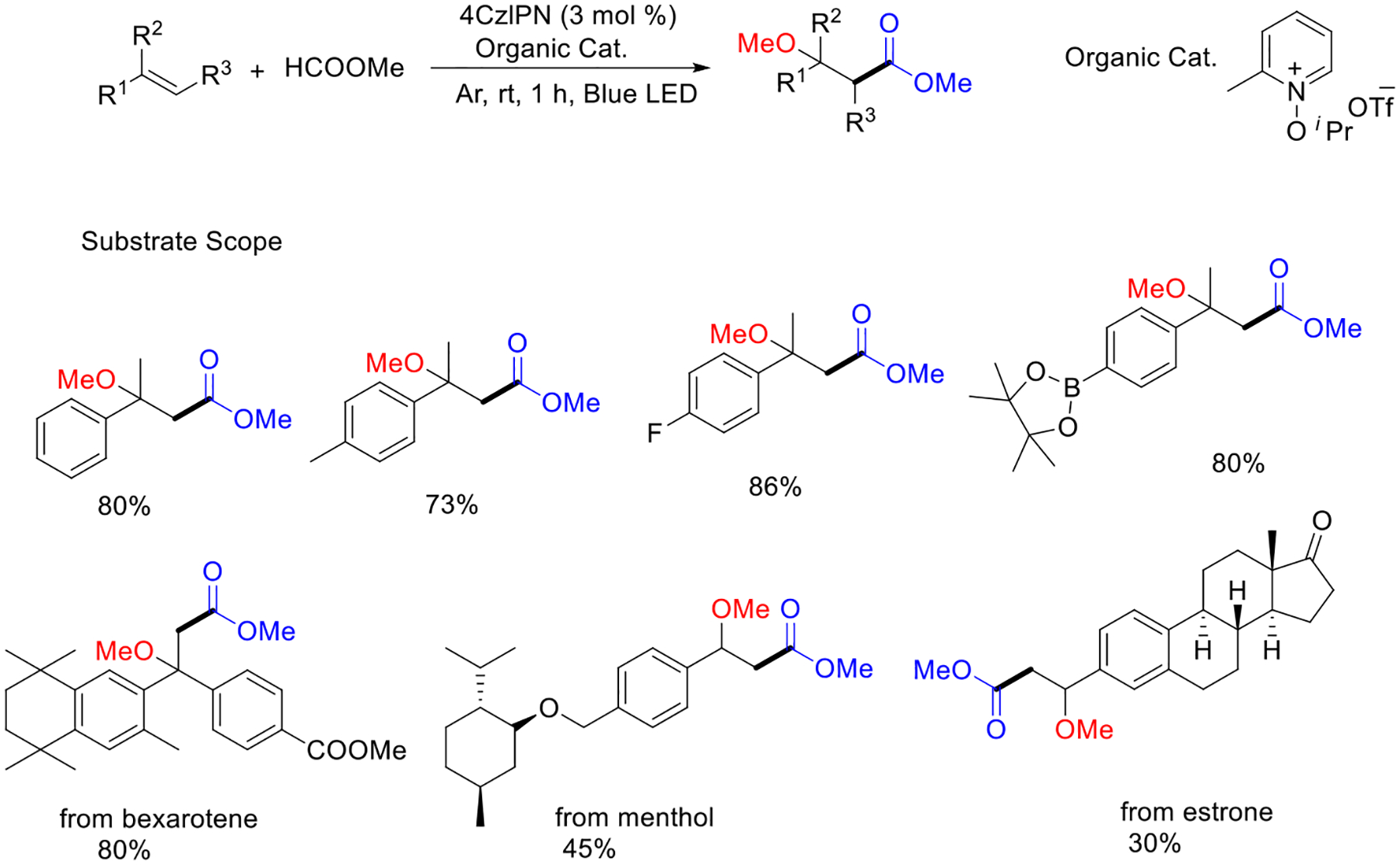
Metal-free divergent alkene functionalization.

At the same time, the Li group demonstrated the alkene functionalization via an in situ-produced oxygen radical species-promoted intermolecular hydrogen atom transfer process by using the organo-photocatalyst 4CzIPN. This method can be applied to realize the carbonylation of natural structural units such as bexarotene, menthol, and estrone (Scheme 29) [78].

**Scheme 30.**
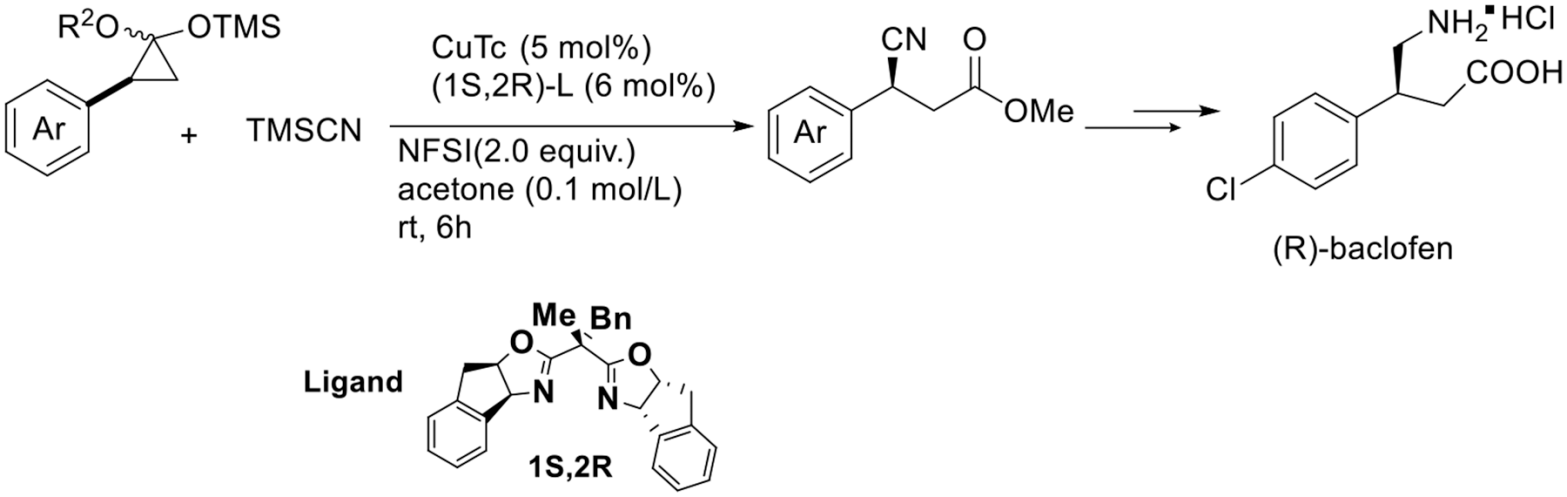
Cu-mediated enantioselective cyanation of cyclopropanols.

In the same year, Liu’s group demonstrated a copper-mediated enantioselective cyanation for the synthesis of enantioenriched β-carbonyl nitriles in outstanding yield. Additionally, the chiral β-cyano propanoic ester formed from the ring opening of cyclo-propanone acetals. The strategy can be applied in the synthesis of GABA receptor agonist (R)-baclofen, which acts as an inhibitory neurotransmitter (Scheme 30) [79].

**Scheme 31.**
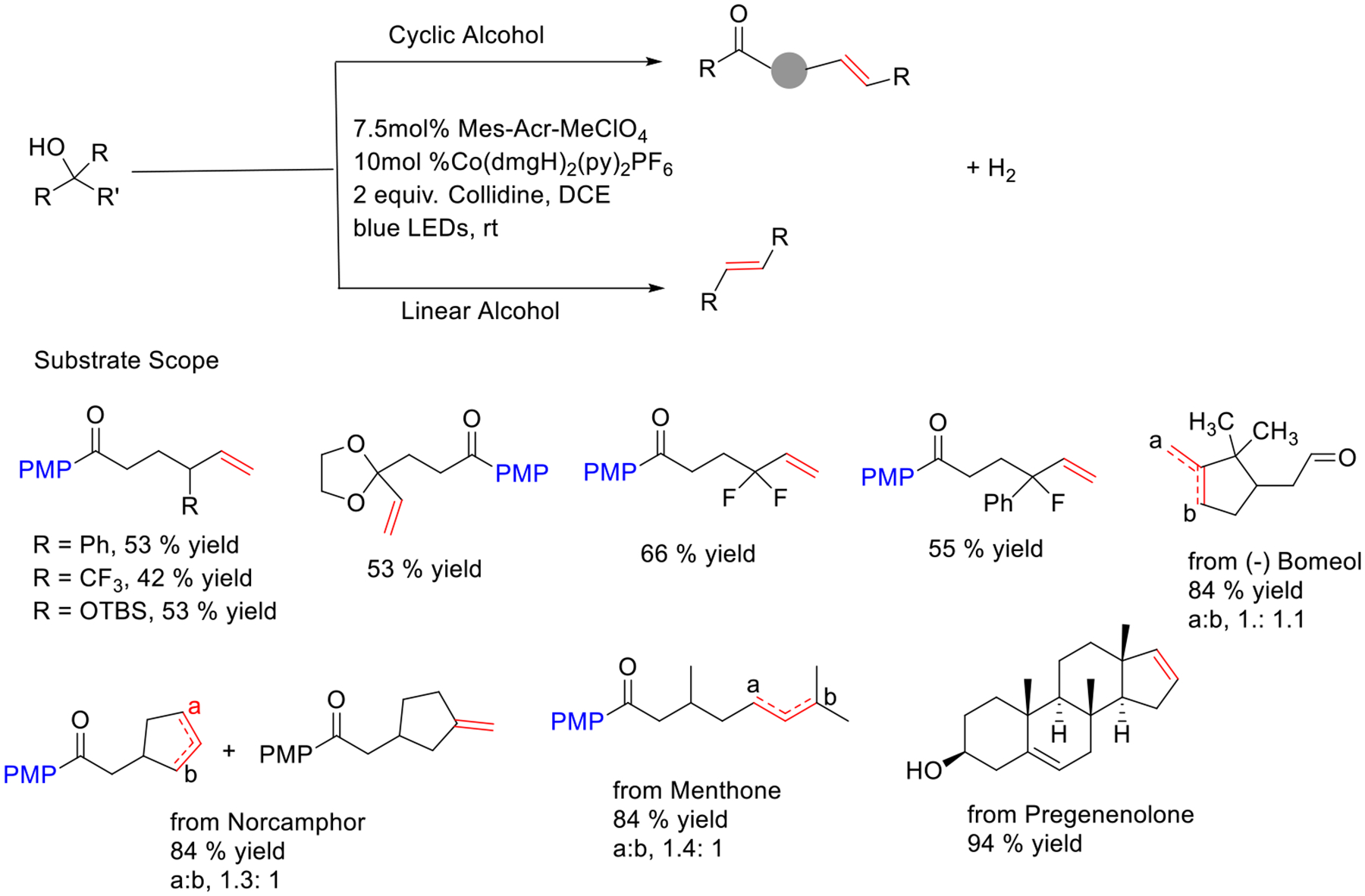
Bioinspired desaturation of alcohols via PCET and cobalt dual catalysis.

Recently, the Rueping group used the photoredox PCET and cobalt synergistic catalytic combination for generating alkoxy radicals from a range of alcohols. This process delivered selective ring-opening of various cyclic alcohols and produced dehydrogenated ketones that are challenging to deliver with current methodologies. Both secondary and tertiary alcohols are well accommodated in this reaction. The strategy can be applied to structures of natural product derivatives such as (−)-Bomeol, Norcamphor, and Pregenenolone (Scheme 31) [80].

**Scheme 32.**
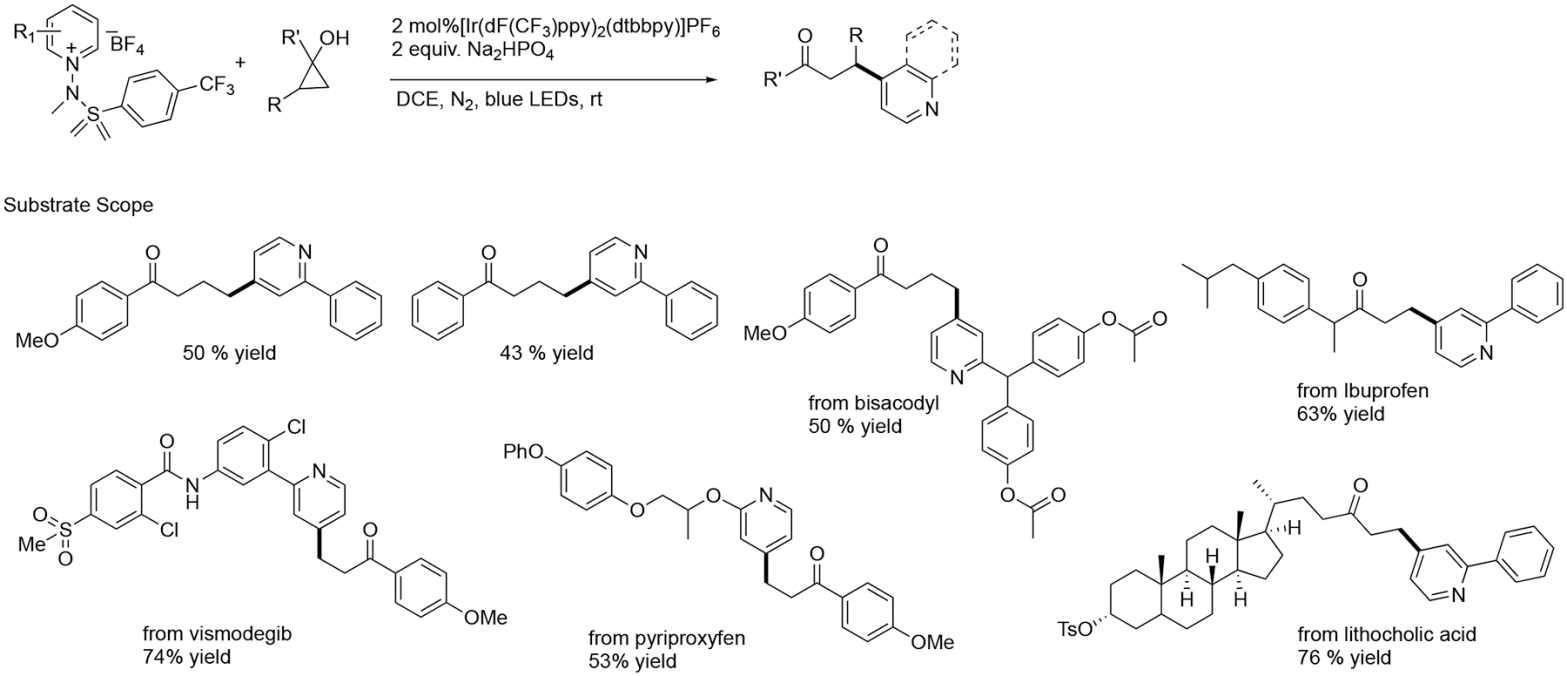
Photoredox promoted selective activation of pyridinium salts.

In addition, the Hong group showed a protocol for site-selective pyridine modification via an alkoxy-centered radical strategy. This approach enabled the concurrent inclusion of pyridyl moiety in architecturally distinct β-carbonyl functionality with outstanding C4 selectivity. This advanced process has been further explored for the selective functionalization of naturally structured designs such as lithocholic acid, ibuprofen, vismodegib, and pyriproxyfen (Scheme 32) [81].

## 3. Application of Alkoxy Radicals in Natural Product Synthesis

**Scheme 33.**
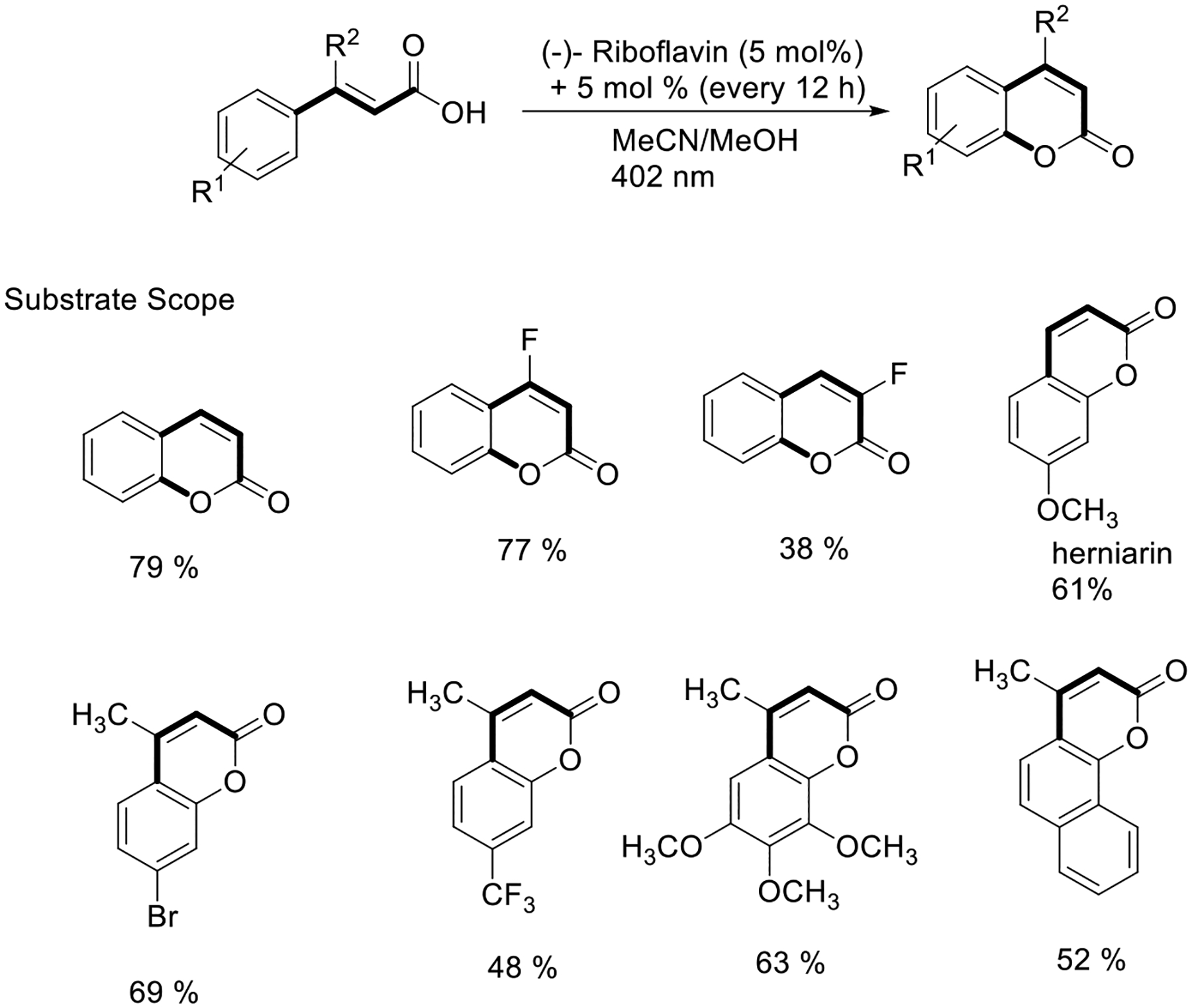
Photocatalysis route to coumarins catalyzed by (−)-riboflavin.

In addition to the development of new synthetic methods, alkoxy radicals have been explored in several elegant syntheses of natural products. In the year 2016, Gilmour’s group showed a novel and practical approach for Z/E configuration alteration and cyclic lactonization of natural product coumarins by employing the organo-catalyst (−)-riboflavin under 402 LED light irradiation, forming in situ alkoxy radical species (Scheme 33) [82].

**Scheme 34.**
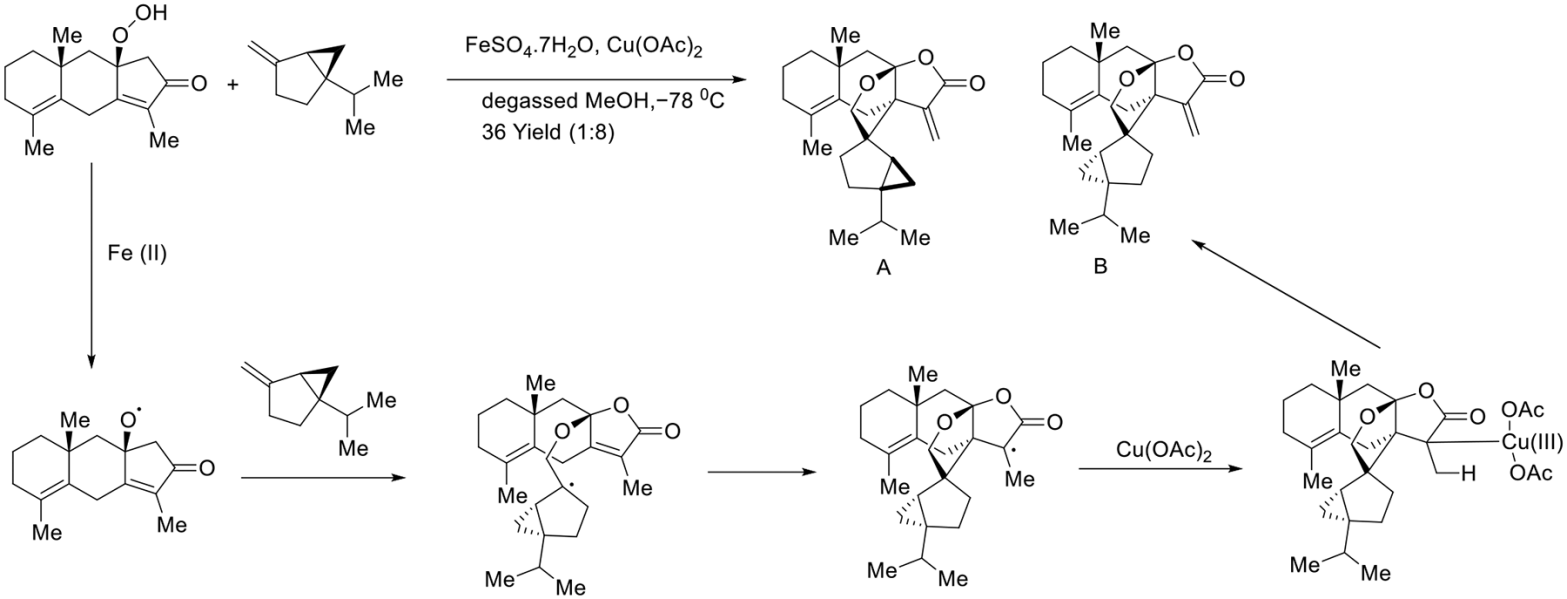
Biomimetic synthesis of hitorins A and B.

In 2019, the Wang group successfully performed a total synthesis of the hitorins using alkoxyl radical chemistry. The interaction between alkoxy intermediate and monoterpene (+)-sabinene provided the corresponding tetrahydrofuran ring of hitorins. The possible mechanism involves generating an alkoxy radical from hydroperoxide using Fe(II) as a reducing agent, which then reacts with (+)-sabinene to produce a carbon-centered radical. The syntheses of hitorin A and B were realized by either radical oxidation by Cu(II) or β-H elimination of intermediate (Scheme 34) [83].

**Scheme 35.**
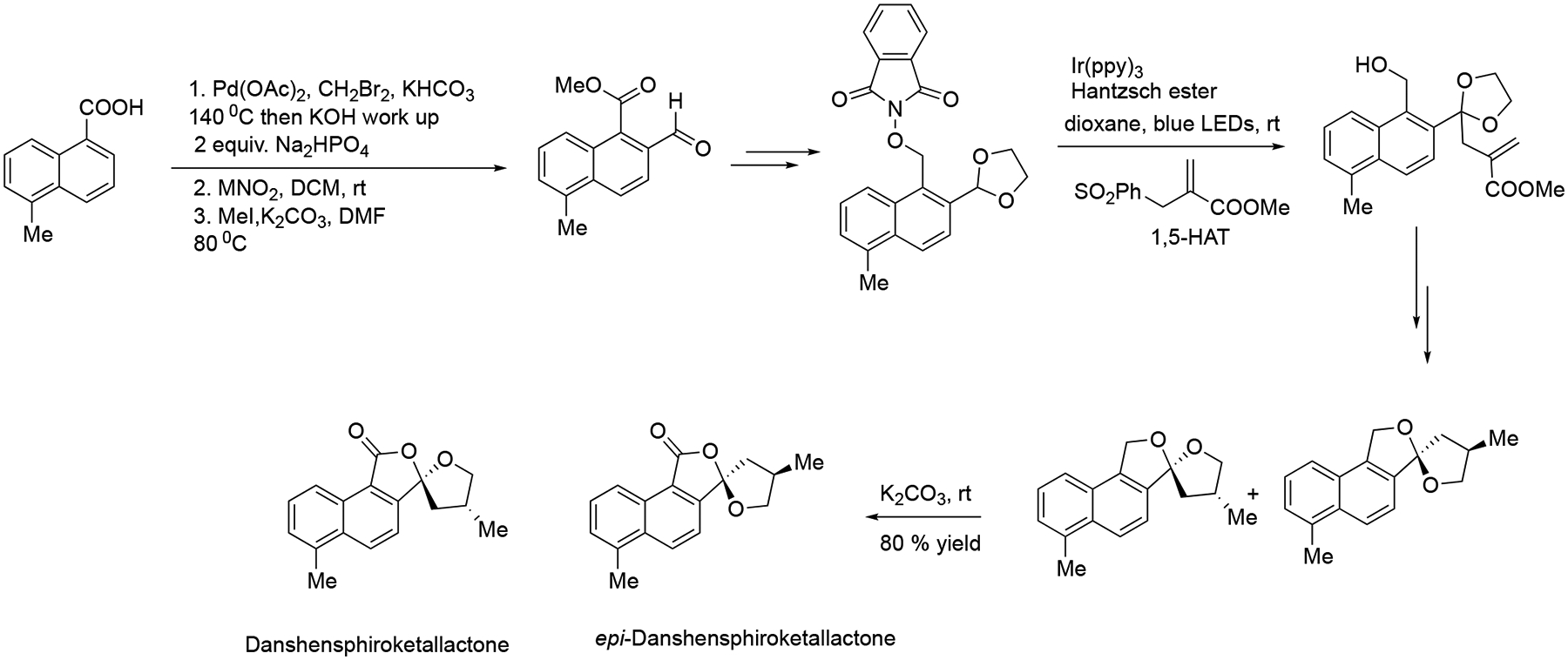
Total synthesis of danshenspiroketallactones.

In the same year, the Smith group designed a novel photoredox approach in order to produce dithianyl and dioxolanyl radical species via the HAT process. The resultant radical intermediates are involved in conjugate additions in order to reach the formal alkylation and allylation. This radical reaction pathway offers an effective way to structure a variety of 1,4,7-polyols or spiro-ketals, which have been applied in the synthesis of danshenspiroketallactones (Scheme 35) [84].

**Scheme 36.**
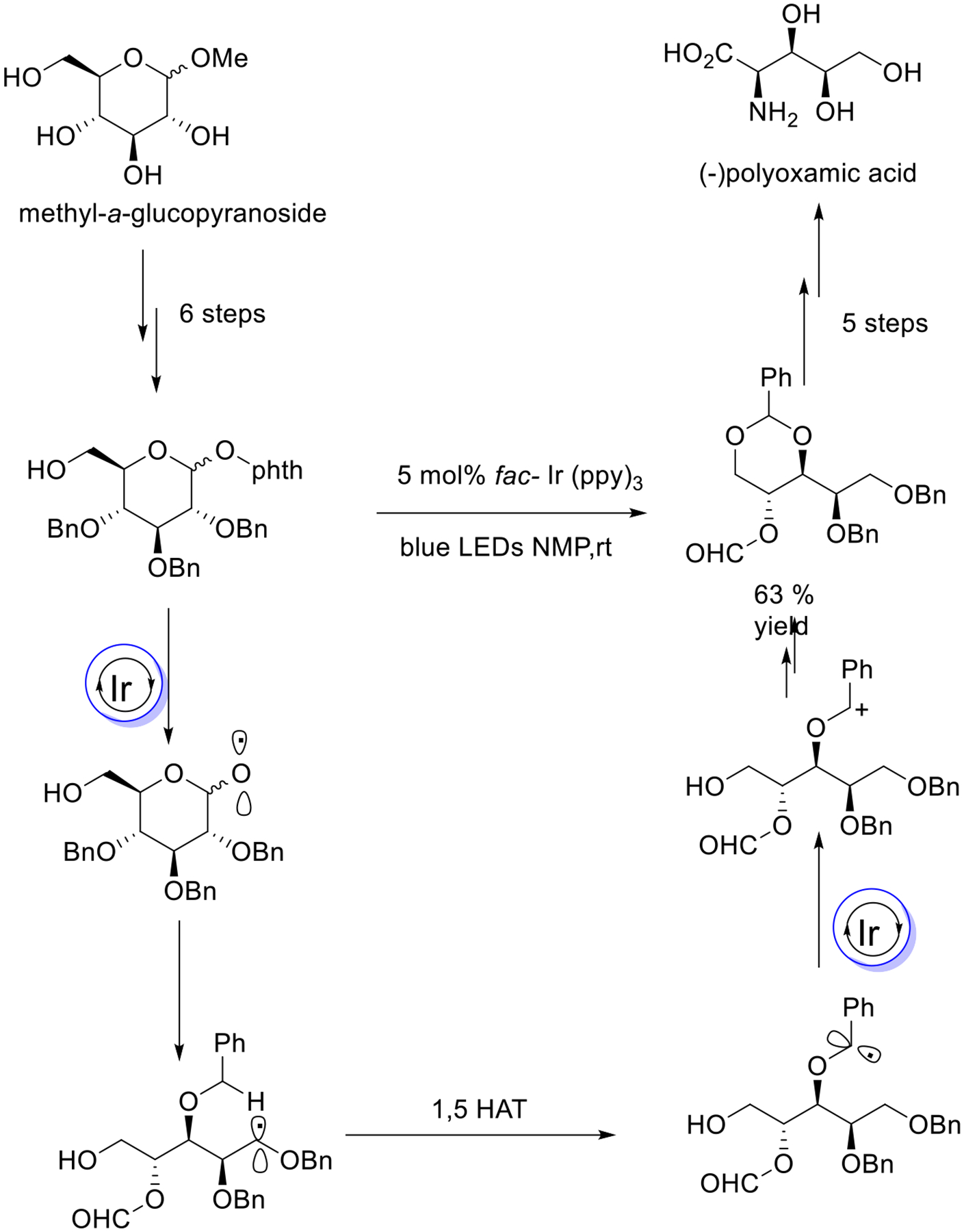
Stepwise synthesis of polyoxamic acid.

A recent example of employing a visible photoredox-promoted strategy for the synthesis of (−)- and (+)-polyoxamic acid from sugar molecules was delivered by the Ohno group. This methodology involves blue light-induced β-fragmentation and a 1,5-HAT strategy to afford the alditol synthesis using modest reaction parameters (Scheme 36) [85].

## 4. Conclusions and Outlook

The effective organic transformation for complex structures demands both a practical approach and the utilization of powerful bond-forming reactions. The effective approaches diminish the unnecessary steps in synthesis and rapidly form new bonds of the target structure. The chemical transformations via radical chemistry deliver an ideal platform for lowering the step for natural product synthesis. This concise review concludes the latest discoveries in the alkoxy radical facilitated organic transformations. Recently, the growing interest in photoredox catalysis broadened the domain of alkoxy radical-promoted organic conversion at an extraordinary pace. These novel and efficient strategies avoid the pre-functionalization of the substrate and stoichiometric reagent and are further explored for late-stage modification in structurally elaborate molecules by alkoxy radical species, including selective activation of the C-H bond, C-N and C-C bond cleavage, and C-O bond shaping. Although alkoxy radical utilization in these activations has broadened the interest in novel chemical transformation, several challenges remain. We anticipate that the discovery of novel alkoxy-radical precursors and practically applicable approaches will further innovate in chemical transformations and lead to an advanced level of natural product synthesis.

## Abbreviations

BDE: Bond dissociation energy
BI: Benziodoxole
CIR: Cyclic iodine(III) reagents
DBAD: Di-tert-butyl azodicarboxylate
DPA: (9,10-diphenylanthracene)
EPR: Electron paramagnetic resonance
HAT: Hydrogen atom transfer
HPLC: High-performance liquid chromatography
HE: Hantzsch ester
LMCT: Ligand-to-metal charge transfer
LED: Light-emitting diode
MLCT: Metal-to-ligand charge transfer
PCET: Proton-coupled electron transfer
PMP: *p*-methoxyphenyl
PIDA: Iodobenzene diacetate
PIFA: [bis(trifluoroacetoxy)iodo]benzene
PPO: Phthaloyl peroxide
PT: Proton Transfer
TBAB: Tetrabutylammonium bromide
4CzIPN: 2,4,5,6-tetra(carbazol-9-yl)benzene-1,3-dicarbonitrile
